# Adolescent intermittent ethanol (AIE) produces lasting, sex-specific changes in rat body fat independent of changes in white blood cell composition

**DOI:** 10.3389/fphys.2024.1285376

**Published:** 2024-01-24

**Authors:** Andrew S. Vore, Paige Marsland, Thaddeus M. Barney, Elena I. Varlinskaya, Justine D. Landin, Kati L. Healey, Sandra Kibble, H. S. Swartzwelder, Lawrence J. Chandler, Terrence Deak

**Affiliations:** ^1^ Developmental Exposure Alcohol Research Center, Binghamton University-SUNY, Binghamton, NY, United States; ^2^ Department of Neurosciences, Charleston Alcohol Research Center, Medical University of South Carolina, Charleston, SC, United States; ^3^ Department of Psychiatry and Behavioral Sciences, Duke University Medical Center, Durham, NC, United States

**Keywords:** rat, cytokine, leukocytes, body composition, adolescent, ethanol, development, immune

## Abstract

Early initiation of alcohol use during adolescence, and adolescent binge drinking are risk factors for the development of alcohol use disorder later in life. Adolescence is a time of rapid sex-dependent neural, physiological, and behavioral changes as well as a period of heightened vulnerability to many effects of alcohol. The goal of the present studies was to determine age-related changes in blood (leukocyte populations) and body composition across adolescence and early adulthood, and to investigate whether adolescent intermittent ethanol (AIE) exposure would alter the trajectory of adolescent development on these broad physiological parameters. We observed significant ontogenetic changes in leukocyte populations that were mirrored by an age-related increase in cytokine expression among mixed populations of circulating leukocytes. Despite these developmental changes, AIE did not significantly alter overall leukocyte numbers or cytokine gene expression. However, AIE led to sex-specific changes in body fat mass and fat percentage, with AIE-exposed male rats showing significantly decreased fat levels and female rats showing significantly increased fat levels relative to controls. These changes suggest that while AIE may not alter overall leukocyte levels, more complex phenotypic changes in leukocyte populations could underlie previously reported differences in cytokine expression. Coupled with long-term shifts in adipocyte levels, this could have long-lasting effects on innate immunity and the capacity of individuals to respond to later immunological and physiological threats.

## Introduction

Alcohol misuse represents a significant public health concern as a leading risk factor contributing to premature death (estimated involvement in 10% of all cases) in individuals aged 15 to 49 (Volkov, 2022; Schuckit, 2009). Binge drinking, defined by NIAAA as a pattern of consumption resulting in blood ethanol concentrations (BECs) of 0.08 g/dL or higher, begins in adolescence (ages 15–18) and peaks in young adulthood (ages 25–34) (Youth Risk Behavior Surveillance system, 2019). Earlier age of drinking initiation as well as higher frequency of binge drinking correlate significantly with increased chances of later development of alcohol use disorder (Grant and Dawson, 1997; Crews et al., 2016). Although binge alcohol consumption during adolescence remains a global health concern, our knowledge about long-lasting effects of adolescent alcohol misuse on the trajectory of physiological and neurobehavioral development is still limited.

Adolescence is a developmental period marked by rapid growth and extensive physiological changes, including changes in body composition, physiology, and sensitivity to drugs. For example, in laboratory rodents, behavioral sensitivity to ethanol is markedly different between adolescents and adults. Adolescent rats are less sensitive than adults to many of the adverse consequences of ethanol, including its aversive (Schramm-Sapyta et al., 2009; Saalfield and Spear, 2016; Gore-Langton et al., 2022), motor-impairing (White et al., 2002; Ramirez and Spear, 2010), and sedative (Little et al., 1996) effects, also demonstrating heightened sensitivity to positive, reinforcing, and stimulatory effects (Pautassi et al., 2008; Varlinskaya et al., 2015). This adolescent-typical pattern of ethanol responsiveness (Spear, 2013) may contribute to the increased levels of alcohol consumption observed in adolescent rats relative to their adult counterparts (Truxell, Molina and Spear, 2007; Spear, 2013). Adolescent rats also appear to be more sensitive than adults to adverse consequences of chronic intermittent ethanol, including increased brain damage (Crews et al., 2000), decreased neurogenesis (Crews et al., 2006), and heightened inflammation (Pascual et al., 2007). Curiously, however, induction of neuroimmune genes after lipopolysaccharide (LPS), ethanol, or acute stress is severely muted in early adolescent rats when compared to adults, suggesting some functional immaturity in adolescent neuroimmune reactivity (Doremus-Fitzwater et al., 2015; Marsland et al., 2022). In addition, binge-like ethanol exposure during adolescence has been shown to produce long-lasting changes in microglial morphology consistent with a dystrophic state (McClain et al., 2011), astrocyte-synaptic proximity (Healey et al., 2020; Healey et al., 2022), dysregulation of glial cells (Nwachukwu et al., 2022), increased neuroimmune gene expression (Crews et al., 2019; Vore et al., 2021), and induction of cytokine expression among circulating lymphocytes (Vore et al., 2017; 2021). Alcohol and its metabolites are also well recognized as having harmful effects on the gastrointestinal tract and liver, with inflammation originating in these areas often contributing to more widespread alcohol-induced organ damage later (Bishehsari et al., 2017). Together, these findings suggest that binge drinking in adolescence may result in heightened vulnerability to alcohol-induced inflammation in both central and peripheral tissue compartments.

While a significant number of studies have investigated the long-term neuroimmune consequences of AIE, fewer have focused upon changes on peripheral markers of inflammation. The majority of the immune system exists outside of the CNS and it is common practice in clinical settings to probe peripheral markers of inflammation to glean insight into CNS health (Chaussabel, Pascual and Banchereau, 2010). One of the simplest methods of assessing these peripheral markers is through analysis of blood cell counts and composition. Blood flow represents an important method of transit for immune cells to reach different sites of action (i.e., lymph nodes), from which antigen-mediated immune responses are derived. These circulating immune cells play a vital role in both the detection of immunological threat and propagation of the immune response and receive direct pharmacological contact with ethanol circulating in the blood. In humans, heavy alcohol consumption patterns are associated with abnormalities in RBC morphology, reduced platelet levels, and reduced WBC levels (Ballard, 1997). While acute alcohol intoxication has been shown to alter monocyte populations, ultimately resulting in a decreased immune response to LPS challenge (Mandrekar et al., 2009; Janicova et al., 2021), chronic alcohol exposure sensitizes monocytes to LPS (Mandrekar et al., 2009). Chronic alcohol consumption also changes lymphocyte function, reducing both B (Sacanella et al., 1998) and T cell (Percival and Sims, 2000) levels as well as altering their activation (see Pasala et al., 2015 for relevant review). While these consequences have often been reported as being reversible following prolonged abstinence, few experiments have directly probed whether binge alcohol consumption during adolescence may produce durable hematological alterations in commonly used animal models.

While individuals with AUD often display greater vulnerability to infection (Szabo and Saha, 2015; Deak et al., 2022), acute ethanol in laboratory rodents has been shown to interfere with leukocyte recruitment after LPS challenge (Saeed et al., 2004). In addition, several studies have reported that ethanol has potentially immunosuppressive effects on monocytes (Chen et al., 1998), lymphocytes (Watson et al., 1988), and neutrophils (Tamura et al., 1998). Prior work has also demonstrated that AIE exposure produced a long-lasting attenuation of the leukocyte-derived cytokine response to either LPS or restraint stress challenge, with these effects being observed exclusively in males (Vore et al., 2017). Whether these changes in immune reactivity reflect AIE-related changes in blood leukocyte composition, however, remains unclear.

One of the critical contributors to inflammatory status of the body is body composition, as both adipocytes (Mohammed-Ali et al., 1998; Coppack, 2001) and skeletal muscles (Peake et al., 2015; Howard et al., 2020) serve as sources of circulating cytokines, termed adipokines and myokines, respectively. Adipose tissue, through specific messenger adipokines, is capable of directly altering immune cell activity. In obese individuals, adipokines have been shown to be upregulated and contribute to inflammation associated with this pathological state (Tilg and Moschen, 2006). Most circulating leukocyte populations contain adipokine receptors (Keustermans et al., 2017) through which direct modulation of immune signaling is possible. In terms of body composition, there are consistent reports that in humans, chronic alcohol contributes to osteoporosis and loss of bone density (Santolaria et al., 2000). However, these effects likely depend on the quantity and regularity of alcohol consumption, with evidence suggesting that low to moderate drinking may increase bone mineral density (Jugdaohsingh et al., 2006), effects that likely reflect bone remodeling (Maurel et al., 2012). While Clayton et al. (2021) showed that chronic drinking among older rats (beginning at 12 months of age) had no effect on bone mineral density, no studies have examined the influence of binge-like ethanol exposures during adolescence, an age when bone size and density expands rapidly (Rauch and Schoenau, 2001), or how AIE influences bone mineral density across the lifespan. Thus, one goal of the present study was to evaluate the influence of AIE and subsequent changes in cytokine function on body composition, including assessments of both adiposity and bone mineral density using a non-invasive procedure (DXA scanning) that permits longitudinal assessments.

The present studies sought to determine long-lasting changes in immune relevant physiology associated with AIE. While several acute and chronic studies have examined the impact of alcohol on blood and body composition, only a few have characterized longitudinal changes that may occur during and after AIE. Thus, there is a great need to evaluate how AIE might influence gross physiological parameters such as circulating leukocyte populations and body composition. To do this, Experiment 1 investigated natural (unchallenged) developmental differences in blood composition and leukocyte levels to better understand how blood composition changes across adolescent development in male and female rats. We hypothesized that leukocyte levels would increase as animals grew older. Experiment 2 was designed to assess immediate effects of AIE during adolescence and to test its long-lasting consequences in adulthood on cytokine gene expression. Blood composition was then analyzed in adulthood to investigate any long-lasting changes in leukocyte populations as a result of AIE. Previously we have shown that male rats with a history of AIE show altered *Il-1β*, *Tnf-α*, *Il-6*, *Iκbα* gene expression in response to restraint stress and LPS challenge (Vore et al., 2017). Subsequently, we hypothesized that AIE would produce long lasting changes in cytokine gene expression paralleled by increase in blood leukocyte levels. To assess the generalizability of these changes across AIE exposure models, Experiment 3 investigated whether adult animals with a history of AIE administered either by intragastric intubation or through vapor inhalation would show similar long-lasting changes in cytokine reactivity to ethanol challenge. We hypothesized that regardless of AIE exposure model, cytokine reactivity to ethanol challenge would be similarly increased by AIE. Finally, Experiment 4 investigated whether AIE would produce long-term changes in body composition capable of persisting into adulthood. We hypothesized that AIE would increase fat mass in both male and female rats. These experiments provide a comprehensive collection of longitudinal, scientifically rigorous assessments of the long-term consequences of AIE on immune-relevant metrics.

## Materials and methods

### General methods

#### Subjects

Experiments 1, 2, and 4 utilized male and female Sprague Dawley rats bred on site at Binghamton University using breeders acquired from Envigo. Rats were weaned on postnatal day (P) 21 and pair-housed with non-littermates. To mitigate litter effects, no more than two offspring from any litter were assigned to a given experimental group. Rats were housed in standard, clear Plexiglas cages with *ad libitum* access to food (LabDiet; 5L0D) and water. Colony conditions were maintained at 22+/− °C on a 12:12 light:dark cycle (lights on at 0700). In all experiments, rats were treated in accordance with Public Health Service (PHS) policy and all experimental protocols were approved by the Institutional Animal Care and Use Committee (IACUC) at Binghamton University.

#### Adolescent intermittent ethanol (AIE) exposure

Published studies using AIE all follow a similar set of ages and intermittent ethanol exposure procedures, often times varying in the doses, timing, and route of administration. Studies reported here used intubation, though we also present data with AIE models from vapor inhalation (see below). Experiments 2 and 4 utilized identical AIE procedures previously shown to produce long-lasting changes in immune function among other physiological markers (Vore et al., 2017; Vore et al., 2021; Vore et al., 2022). Beginning in early adolescence (P30 ± 2 days), rats received once daily intragastric (i.g.) intubation of 4.0 g/kg ethanol or an equivalent volume of vehicle (tap water). Ethanol solutions were prepared daily (20%, v/v) using 95% ethanol stock diluted in tap water. Body weights were taken each day at least 60 min prior to intubation. Rats would then receive three consecutive days of daily intubations, followed by a 2-day period of abstinence during which rats were left unmanipulated in their home cage. This 5 day “cycle” was repeated 4 times for a total of 20 days of manipulation and 12 ethanol exposures. In Experiments 2 and 4, a non-manipulated control group was also included that were weighed on the same days as AIE and Vehicle groups but did not receive any intubations.

#### Ethanol injection

In Experiments 2 and 3, all rats received a 2.0 g/kg intraperitoneal (i.p.) ethanol injection on P74. This dose was calculated using animal weights collected on P73 and a 20% ethanol solution (v/v) was prepared fresh using 95% ethanol stock diluted in physiological saline.

#### Blood ethanol concentrations

In Experiment 2, blood ethanol concentrations were evaluated following P74 ethanol challenge using an Analox AM-1 alcohol analyzer (Analox Instruments, Lunenburg, MA). The machine was calibrated using a 100 mg/dL industry standard. Accuracy of the machine was rechecked by re-reading of the standard every 15 samples.

#### Blood processing for cytokine RT-PCR

In Experiments 2 and 3, approximately 200 uL of whole blood was collected from the tail into RNAse-free 2.0 mL microcentrifuge tubes when rats were at P29, P44, P73, and P74. Samples were maintained at 4°C on ice for no longer than 30 min, then serum was separated through refrigerated centrifugation and stored at −20°C until later analysis. The remaining blood pellets were stored at −80°C until analysis using the procedures as previously described (Vore et al., 2017). Blood pellets collected at Duke and MUSC (as noted below) were shipped on dry-ice to Binghamton University where they were returned to a −80°C freezer until processed for analysis of blood cytokine gene expression using RT-PCR.

#### Blood processing for complete blood counts

In Experiments 1 and 2, blood for CBC analysis was collected using cardiac puncture. Whole blood was collected into an EDTA-containing vacutainer and immediately placed onto ice until analyzed.

#### Reverse-transcription polymerase chain reaction

All RT-PCR was performed using procedures previously described (Vore et al., 2017). RNA was extracted from the blood using Trizol RNA reagent (Invitrogen; 15,596,026) through use of a TissueLyser and 5 mm stainless steel beads (Qiagen; 69,997). Total RNA was extracted and purified using RNeasy mini columns (Qiagen; 74,106) following manufacturer’s instructions. The purity and concentration of the extracted RNA was quantified using a NanoDrop system (Thermoscientific). cDNA was then synthesized using a Quantitect reverse transcription kit (Qiagen; 205,314) and 1.0 μg of RNA per reaction. This cDNA was then diluted in a 1:2 ratio in RNAse free water and RT-PCR was run using a CFX-384 detection system (Bio-Rad). All primer sequences used can be found in Supplementary Table S1.

#### Statistical analysis

**FIGURE 1:**
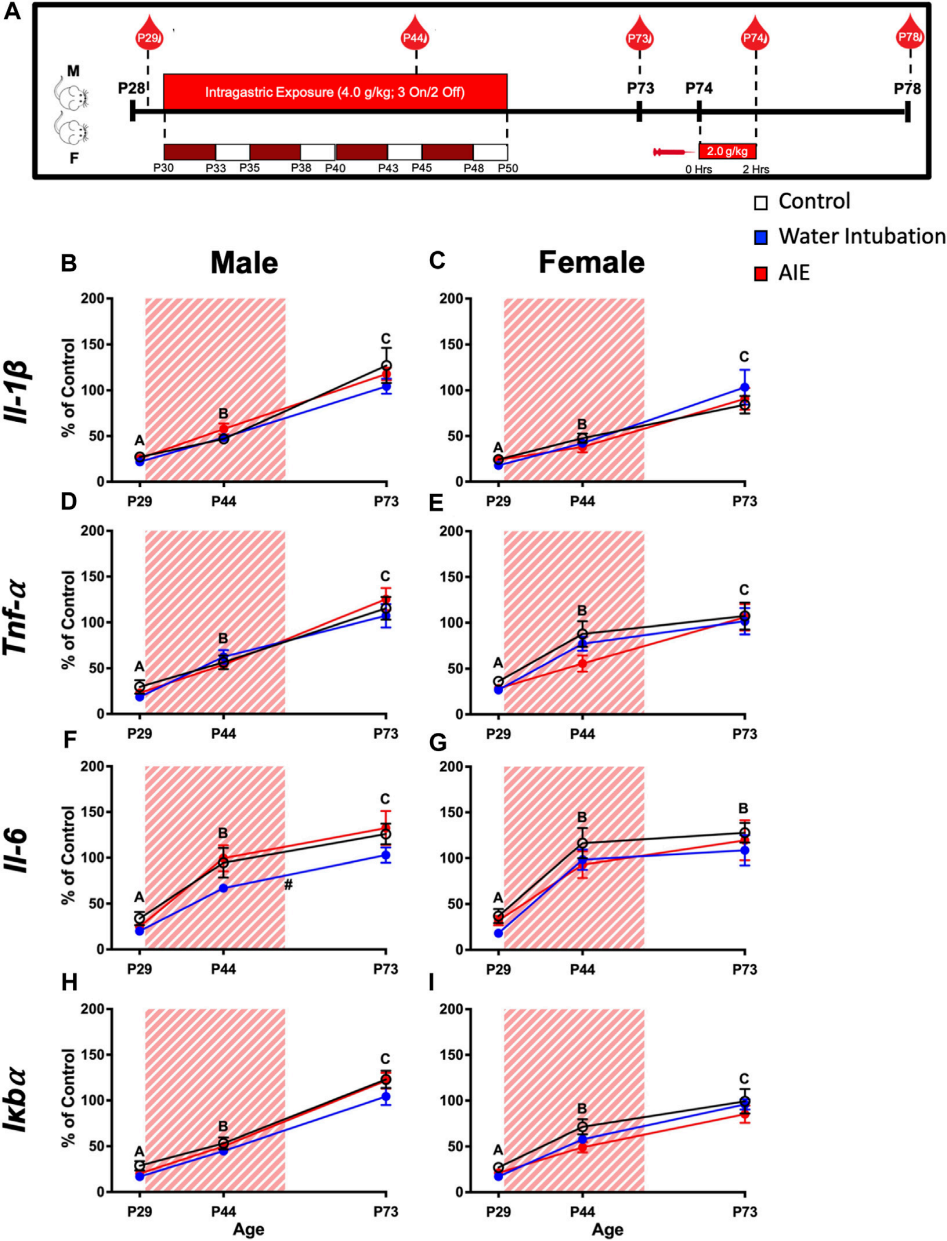
Exp. 2 Timeline and Blood Cytokine Gene Expression Across Development Prior to Ethanol Challenge. Schematic depiction reflecting the timeline of adolescent intermittent ethanol exposure, adult ethanol challenge, and when blood samples were collected **(A)**. Significant main effects of age were reported in both male and female rats in IL-1β, *Tnf-α*, *Il-6*, and *Iκbα* with blood cytokine gene expression increasing as rats aged **(B–I)**. Male rats with a history of water intubation showed significantly decreased *Il-6* expression relative to comparators **(F)**. Significant (*p* < 0.05) main effects of age are denoted by letters **(A-C)**. Significant (*p* < 0.05) main effects of adolescent exposure are denoted by #. All data is represented as means and SEMs. All data is represented relative to the ultimate control group, the P73 water intubation timepoint.

**FIGURE 2:**
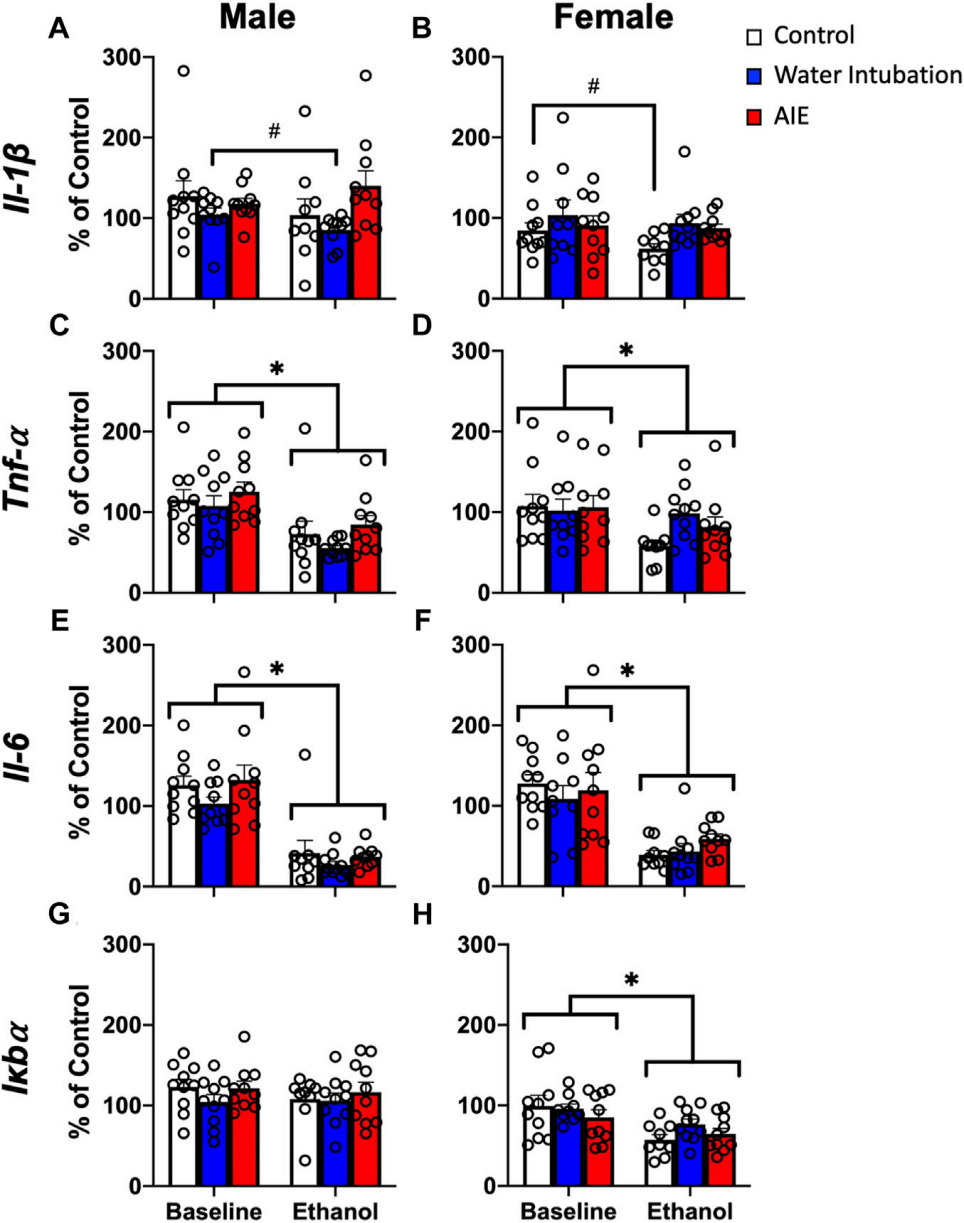
Exp. 2 Adult Blood Cytokine Gene Expression after Ethanol Challenge. Water-exposed controls had significantly lower *Il-1β* expression than non-exposed and AIE-exposed males **(A)**. Both water- and AIE-exposed showed significantly higher *Il-1β* expression than non-exposed control females **(B)**. Ethanol challenge significantly reduced *Tnf-α* and *Il-6* gene expression in both male and female rats **(C–F)**. Ethanol challenge had no effect on male *Iκbα* **(G)** and significantly reduced *Iκbα* expression in female rats **(H)**. Significant (*p* < 0.05) main effects of adolescent exposure are denoted by #. Significant (*p* < 0.05) main effects of ethanol challenge are denoted by *. All data is represented as means and SEMs. All data is represented relative to the ultimate control group, the baseline (P73) water intubation timepoint. The baseline condition is prior to ethanol challenge whereas the ethanol condition is 2 h after i.p. ethanol injection.

**FIGURE 3:**
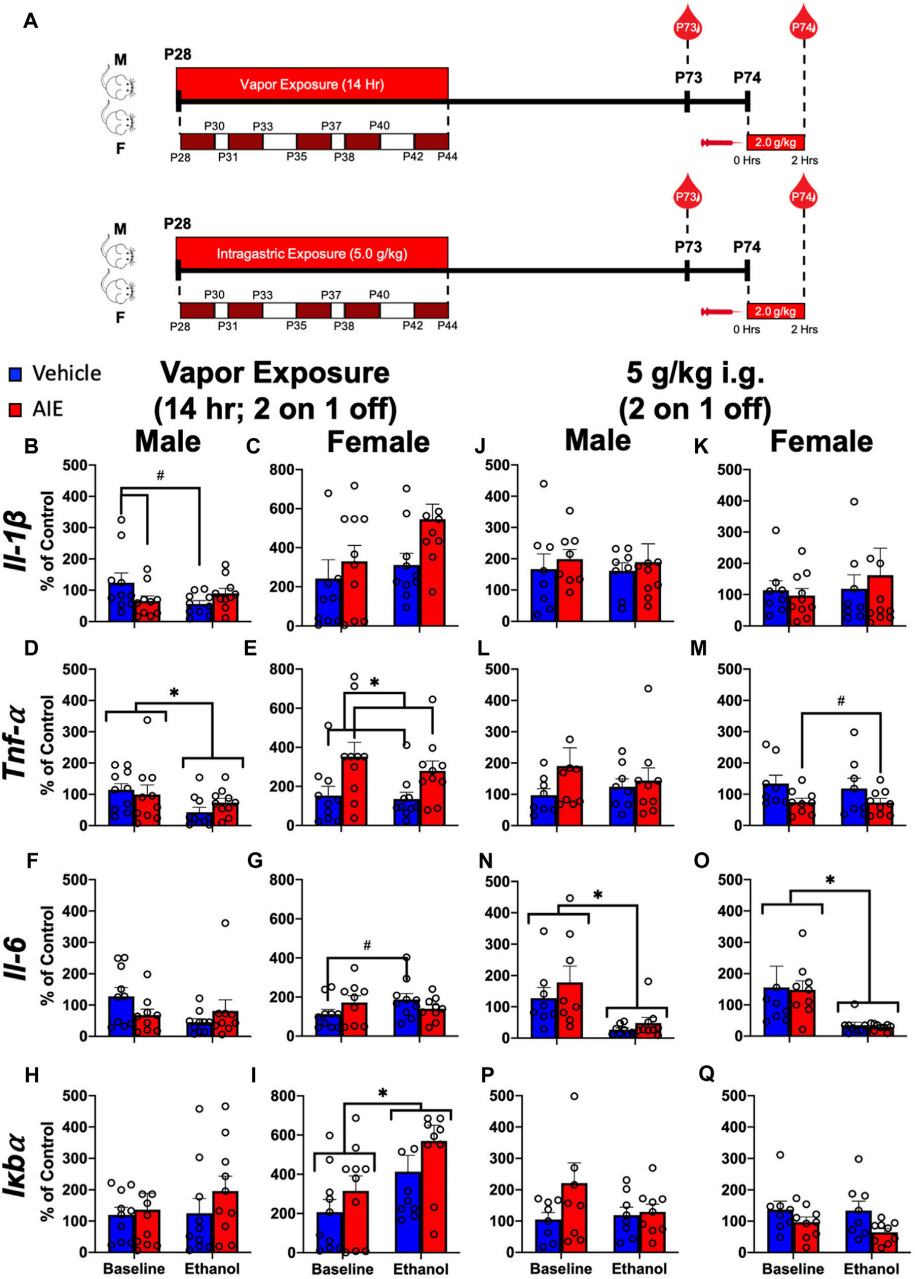
Exp. 3 Effect of Different AIE Models on Adult Blood Cytokine Gene Expression after Ethanol Challenge. Schematic depiction reflecting the timeline of adolescent intermittent ethanol exposure and adult ethanol challenge **(A)**. Male rats with a history of vapor AIE displayed significantly lower *Il-1β* expression relative to air-exposed controls at baseline **(B)**. No such differences were observed in female *Il-1β* expression **(C)**. Adult ethanol challenge significantly decreased *Tnf-α* levels relative to baseline in male rats regardless of adolescent vapor exposure **(D)**. Female rats with a history of vapor AIE displayed significantly higher *Tnf-α* levels relative to air-exposed controls **(E)**. While no significant differences in *Il-6* expression were noted in male rats after vapor exposure **(F)** a significant increase in *Il-6* after adult ethanol challenge evident in air-exposed controls, was not observed in females with an AIE history **(G)**. No significant changes in *Iκbα* expression were observed in male rats regardless of vapor exposure or challenge **(H)**. Finally, adult ethanol challenge significantly increased *Iκbα* expression relative to baseline in female rats **(I)**. No significant changes in *Il-1β* expression were observed after adolescent gavage or adult challenge in either male or female rats **(J and K)**. While no changes in *Tnf-α* were noted in male rats **(L)**, females with a history of gavage AIE showed significantly lower *Tnf-α* levels than water-exposed control females **(M)**. Following adult ethanol challenge, both male **(N)** and female **(O)** rats showed significantly decreased *Il-6* levels relative to baseline. No significant differences in *Iκbα* expression were reported in either male **(P)** or female **(Q)** rats. Significant (*p* < 0.05) interactions are denoted by #. Significant (*p* < 0.05) main effects are denoted by *. All data is represented as means and SEMs. All data is represented relative to the ultimate control group, the baseline (P73) air-exposed or water intubation timepoint. The baseline condition is prior to ethanol challenge whereas the ethanol condition is 2 h after i. p. ethanol injection.

**FIGURE 4:**
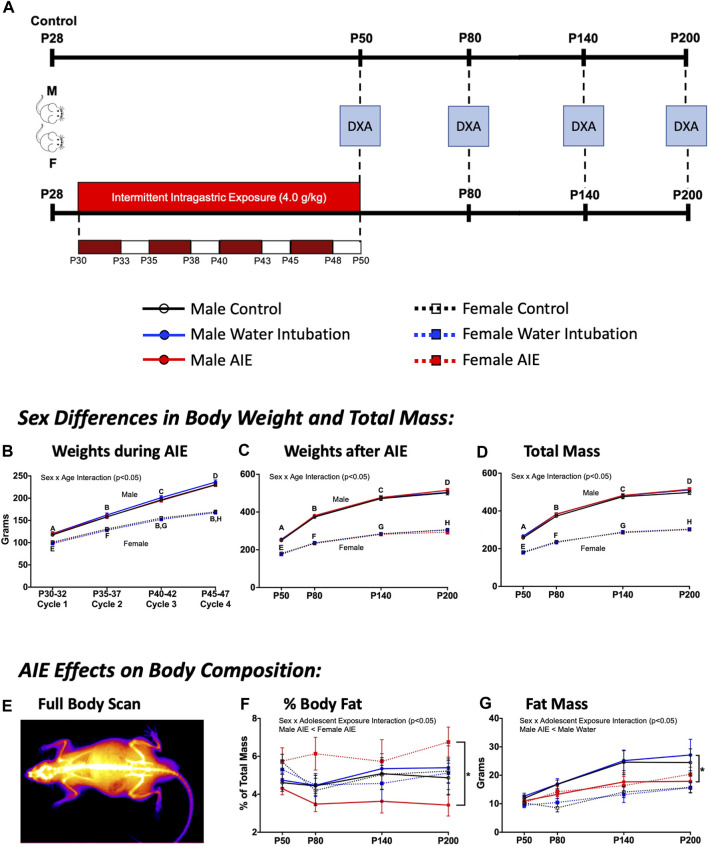
Exp. 4 Changes in Animal Body Composition after AIE. Schematic depiction reflecting the timeline of adolescent intermittent ethanol exposure and when DXA scans were collected **(A)**. Throughout AIE, normal increases in body weight were observed with no significant differences resulting from exposure condition **(B)**. Similar increases in body weight across age were observed when measured using scales **(C)** as well as predicted mass by DXA scanner **(D)**, again no significant differences were observed as a result of exposure condition. A representative heatmap of a DXA scanner image showing high density anatomy like bone (white) and lower density anatomy like fat/muscle (orange-purple) **(E)**. Male rats with a history of AIE showed significantly reduced body fat percentages relative to AIE-exposed females across time **(F)**. AIE-exposed male rats also had significantly lower fat mass than water-exposed controls **(G)**. Significant (*p* < 0.05) sex × age interactions differences between groups are denoted by different letters. Significant (*p* < 0.05) sex by adolescent exposure interactions are denoted by *. All data is represented as means and SEMs.

All data were analyzed using either GraphPad Prism (GraphPad Software, Boston, Massachusetts, United States) or Statistica (TIBCO Data Science; Palo Alto, California, United States) using an ANOVA design that matched the design for each experiment as described below. All data were tested for normality using the Kolmogorov-Smirnov test to ensure normally distributions prior to ANOVA analysis. In cases where significant interactions were achieved, *post hoc* comparisons using Tukey’s HSD test were used to clarify group differences. Criterion for rejection of the null hypothesis was at *p* < 0.05 for all statistical tests. Due to the large number of dependent variables assessed in these studies, key findings from individual experiments are presented in graphical form (Figures 1–4), Tables 1–4 show means ( ± SEM) for large datasets, and Supplemental Tables were used to report statistical parameters corresponding to Tables 1–4.

**TABLE 1:** Experiment 1: Blood Composition Analysis Across Development Means and SEMs for all measured blood composition dependent variables in Experiment 1. Superscript letters indicate *post hoc* differences when a significant sex × age interaction was observed. Main effects are reported in the far right column.

		P28	P45	P90	Significant main effects
Red Blood Cell (100,000 Cells/uL)	Male	5.52 ± 0.25^A^	6.44 ± 0.10^B^	7.95 ± 0.11^C^	Sex: None
	Female	5.60 ± 0.15^A^	6.67 ± 0.06^B^	7.36 ± 0.11^D^	Age: P28 < P45 < P90
Hemoglobin (g/dL)	Male	11.52 ± 0.41^A^	13.53 ± 0.23^B^	15.18 ± 0.16^C^	Sex: M > F
	Female	11.65 ± 0.26^A^	13.60 ± 0.17^B^	13.70 ± 0.12^B,D^	Age: P28 < P45 < P90
Hematocrit (%)	Male	38.83 ± 1.62^A^	43.29 ± 0.92^B^	46.33 ± 0.43^C^	Sex: M > F
	Female	37.88 ± 1.22^A^	42.22 ± 0.49^B^	41.90 ± 0.38^B,D^	Age: P28 < P45/P90
Mean Corpuscular Volume (fL)	Male	70.17 ± 0.70	67.00 ± 0.62	58.22 ± 0.65	Sex: M > F
	Female	67.75 ± 0.62	63.44 ± 0.63	56.90 ± 0.82	Age: P28 > P45 > P90
Mean Corpuscular Hemoglobin (pg)	Male	20.83 ± 0.31	21.00 ± 0.22	19.06 ± 0.13	Sex: M > F
	Female	20.75 ± 0.16	20.22 ± 0.15	18.70 ± 0.37	Age: P28,P45 > P90
Mean Corpuscular Hemoglobin Concentration (g/dL)	Male	29.83 ± 0.31	31.29 ± 0.18	32.83 ± 0.22	Sex: M < F
	Female	30.75 ± 0.41	32.22 ± 0.15	32.80 ± 0.29	Age: P28 < P45 < P90
Red Blood Cell Distribution Width (µM)	Male	19.42 ± 0.81	13.76 ± 0.38	11.39 ± 0.19	Sex: M > F
	Female	18.10 ± 0.65	12.93 ± 0.20	11.02 ± 0.19	Age: P28 > P45 > P90
Platelets (1,000 Cells/µL)	Male	796.33 ± 80.43	1121.29 ± 27.93	828.11 ± 27.00	Sex: M > F
	Female	762.88 ± 62.59	889.00 ± 88.65	677.80 ± 41.76	Age: P45 > P28,P90
Mean Platelet Volume (fL)	Male	9.00 ± 0.27	7.03 ± 0.07	6.98 ± 0.13	Sex: None
	Female	8.79 ± 0.23	7.24 ± 0.25	7.00 ± 0.11	Age: P28 > P45,P90
White Blood Cell (1,000 Cells/µL)	Male	6.37 ± 0.53	6.94 ± 1.25	5.04 ± 0.50	Sex: None
	Female	5.01 ± 0.34	5.83 ± 0.58	4.38 ± 0.68	Age: P45 > P90
Absolute Neutrophil Count (1,000 Cells/µL)	Male	0.65 ± 0.07	0.64 ± 0.08	0.76 ± 0.07	Sex: M > F
	Female	0.54 ± 0.05	0.63 ± 0.07	0.40 ± 0.08	Age: None
Relative Neutrophil (%)	Male	10 ± 1.0^A^	11 ± 2.0^A^	16 ± 1.0^B^	Sex: None
	Female	11 ± 1.0^A^	11 ± 1.0^A^	9 ± 1.0^A^	Age: None
Absolute Eosinophil Count (1,000 Cells/µL)	Male	0.05 ± 0.02	0.04 ± 0.02	0.11 ± 0.02	Sex: None
	Female	0.05 ± 0.02	0.07 ± 0.02	0.07 ± 0.02	Age: None
Relative Eosinophil (%)	Male	1.0 ± 0.30	1.0 ± 0.20	2.0 ± 0.30	Sex: None
	Female	1.0 ± 0.40	1.0 ± 0.30	2.0 ± 0.40	Age: P28,P45 < P90
Absolute Monocyte Count (1,000 Cells/µL)	Male	0.27 ± 0.04	0.19 ± 0.04	0.10 ± 0.02	Sex: None
	Female	0.16 ± 0.04	0.14 ± 0.02	0.04 ± 0.02	Age: P28,P45 > P90
Relative Monocyte (%)	Male	4.0 ± 0.40	3.0 ± 0.30	2.0 ± 0.30	Sex: None
	Female	3.0 ± 0.60	3.0 ± 0.20	0.60 ± 0.30	Age: P28 > P45 > P90
Absolute Lymphocyte Count (1,000 Cells/µL)	Male	5.27 ± 0.42	6.00 ± 1.15	4.04 ± 0.44	Sex: None
	Female	4.18 ± 0.29	4.91 ± 0.50	3.81 ± 0.59	Age: P45 > P90
Relative Lymphocyte (%)	Male	84.0 ± 1.0^A^	86.0 ± 2.0^A^	80.0 ± 1.0^B^	Sex: M < F
	Female	85.0 ± 1.0^A^	85.0 ± 1.0^A^	89.0 ± 1.0^C^	Age: None
Absolute Basophil Count (1,000 Cells/µL)	Male	N/A	N/A	N/A	Sex: None
	Female	N/A	N/A	N/A	Age: None
Relative Basophil (%)	Male	N/A	N/A	N/A	Sex: None
	Female	N/A	N/A	N/A	Age: None

**TABLE 2:** Experiment 2: Blood Ethanol Concentrations after P74 Ethanol Challenge Means and SEMs for blood ethanol concentrations after adult ethanol challenge in Experiment 2.

		*Ethanol*		
		*Control*	*Water Intubation*	*AIE*
*BECs*	*Male*	144.88 ± 2.69	150.09 ± 3.02	141.43 ± 2.65
	*Female*	119.70 ± 4.22	115.70 ± 2.60	113.64 ± 3.69

**TABLE 3:** Experiment 2: Blood Composition Analysis Means and SEMs for all measured blood composition dependent variables in Experiment 2. Main effects are reported in the far right column.

		Control	Water intubation	AIE	Significant main effects
Red Blood Cell (100,000 Cells/uL)	Male	7.57 ± 0.12	7.63 ± 0.15	7.81 ± 0.08	Sex: M > F
	Female	7.00 ± 0.12	7.25 ± 0.13	6.97 ± 0.14	Adolescent Exposure: None
Hemoglobin (g/dL)	Male	15.00 ± 0.22	14.98 ± 0.23	15.41 ± 0.17	Sex: M > F
	Female	13.93 ± 0.17	14.06 ± 0.16	13.87 ± 0.18	Adolescent Exposure: None
Hematocrit (%)	Male	44.30 ± 0.92	44.13 ± 1.08	45.78 ± 0.55	Sex: M > F
	Female	40.75 ± 0.41	41.00 ± 0.46	40.00 ± 0.93	Adolescent Exposure: None
Mean Corpuscular Volume (fL)	Male	58.50 ± 0.65	57.75 ± 0.56	58.56 ± 0.48	Sex: None
	Female	58.38 ± 0.56	56.88 ± 0.93	57.4 ± 0.48	Adolescent Exposure: None
Mean Corpuscular Hemoglobin (pg)	Male	19.90 ± 0.10	19.50 ± 0.19	19.78 ± 0.15	Sex: None
	Female	20.00 ± 0.27	19.50 ± 0.42	20.00 ± 0.24	Adolescent Exposure: None
Mean Corpuscular Hemoglobin Concentration (g/dL)	Male	33.90 ± 0.38	34.13 ± 0.44	33.89 ± 0.11	Sex: None
	Female	34.25 ± 0.16	34.13 ± 0.23	34.44 ± 0.38	Adolescent Exposure: None
Red Blood Cell Distribution Width (µM)	Male	11.00 ± 0.10	11.05 ± 0.17	10.81 ± 0.05	Sex: None
	Female	11.61 ± 0.38	10.84 ± 0.06	11.11 ± 0.18	Adolescent Exposure: None
Platelets (1,000 Cells/µL)	Male	999.60 ± 64.78	986.50 ± 50.81	1077.56 ± 33.03	Sex: None
	Female	1007.25 ± 60.25	1178.38 ± 64.17	1032.56 ± 115.46	Adolescent Exposure: None
Mean Platelet Volume (fL)	Male	6.75 ± 0.19	6.93 ± 0.20	6.74 ± 0.09	Sex: None
	Female	6.61 ± 0.05	6.54 ± 0.06	6.83 ± 0.11	Adolescent Exposure: None
White Blood Cell (1,000 Cells/µL)	Male	6.04 ± 0.60	5.86 ± 0.66	6.27 ± 0.36	Sex: None
	Female	5.39 ± 0.44	4.86 ± 0.44	5.26 ± 0.64	Adolescent Exposure: None
Absolute Neutrophil Count (1,000 Cells/µL)	Male	0.76 ± 0.10	0.83 ± 0.07	0.92 ± 0.09	Sex: M > F
	Female	0.54 ± 0.07	0.53 ± 0.07	0.68 ± 0.08	Adolescent Exposure: None
Relative Neutrophil (%)	Male	13.71 ± 1.71	15.17 ± 1.52	14.65 ± 0.81	Sex: M > F
	Female	9.92 ± 0.65	10.82 ± 1.00	13.99 ± 1.52	Adolescent Exposure: None
Absolute Eosinophil Count (1,000 Cells/µL)	Male	0.07 ± 0.02	0.09 ± 0.01	0.10 ± 0.00	Sex: None
	Female	0.10 ± 0.00	0.09 ± 0.01	0.09 ± 0.01	Adolescent Exposure: None
Relative Eosinophil (%)	Male	1.60 ± 0.61	1.47 ± 0.26	1.65 ± 0.11	Sex: None
	Female	1.95 ± 0.15	1.79 ± 0.29	2.27 ± 0.79	Adolescent Exposure: None
Absolute Monocyte Count (1,000 Cells/µL)	Male	0.11 ± 0.02	0.14 ± 0.03	0.14 ± 0.02	Sex: None
	Female	0.11 ± 0.01	0.13 ± 0.03	0.11 ± 0.02	Adolescent Exposure: None
Relative Monocyte (%)	Male	1.72 ± 0.28	2.15 ± 0.43	2.29 ± 0.21	Sex: None
	Female	2.11 ± 0.14	2.52 ± 0.28	1.94 ± 0.31	Adolescent Exposure: None
Absolute Lymphocyte Count (1,000 Cells/µL)	Male	5.03 ± 0.52	4.75 ± 0.56	5.09 ± 0.29	Sex: None
	Female	4.61 ± 0.36	4.11 ± 0.34	4.36 ± 0.54	Adolescent Exposure: None
Relative Lymphocyte (%)	Male	82.97 ± 2.14	81.21 ± 1.25	81.41 ± 0.72	Sex: None
	Female	86.02 ± 0.77	84.88 ± 1.14	81.80 ± 1.99	Adolescent Exposure: None
Absolute Basophil Count (1,000 Cells/µL)	Male	N/A	N/A	N/A	Sex: None
	Female	N/A	N/A	N/A	Adolescent Exposure: None
Relative Basophil (%)	Male	N/A	N/A	N/A	Sex: None
	Female	N/A	N/A	N/A	Adolescent Exposure: None

**TABLE 4:** Experiment 4: DXA Scanner Analyses. Means and SEMs for all measured DXA Scanner dependent variables in Experiment 4. Superscript letters indicate *post hoc* differences when a significant sex × age interaction was observed. Main effects are reported in the far right column.

		P50			P80			P140			P200			Significant main effects
		Control	Water intubation	AIE	Control	Water intubation	AIE	Control	Water intubation	AIE	Control	Water intubation	AIE	
Bone Mineral Content (g)	Male	5.68 ± 0.10^A^	5.91 ± 0.15^A^	5.66 ± 0.13^A^	9.83 ± 0.18^B^	10.04 ± 0.18^B^	9.81 ± 0.12^B^	13.46 ± 0.23^C^	13.62 ± 0.28^C^	13.61 ± 0.26^C^	14.65 ± 0.18^D^	14.99 ± 0.28^D^	15.14 ± 0.24^D^	Sex: M > F Age: P50 < P80 < P140 < P200 Adolescent Exposure: None
	Female	4.88 ± 0.09^E^	4.84 ± 0.07^E^	4.88 ± 0.09^E^	7.40 ± 0.16^F^	7.18 ± 0.22^F^	7.26 ± 0.12^F^	9.68 ± 0.21^B^	9.90 ± 0.12^B^	9.85 ± 0.15^B^	10.64 ± 0.15^G^	10.61 ± 0.15^G^	10.70 ± 0.17^G^	
Area (cm^2^)	Male	42.89 ± 0.98^A^	45.73 ± 1.06^A^	42.62 ± 1.40^A^	58.92 ± 1.78^B^	61.00 ± 0.60^B^	58.54 ± 1.07^B^	69.84 ± 1.42^C^	69.78 ± 1.81^C^	70.15 ± 1.32^C^	75.13 ± 0.70^D^	76.80 ± 1.02^D^	76.32 ± 1.03^D^	Sex: M > F Age: P50 < P80 < P140 < P200 Adolescent Exposure: None
	Female	37.35 ± 0.72^E^	38.31 ± 0.73^E^	38.13 ± 0.99^E^	46.06 ± 0.77^A^	45.47 ± 0.77^A^	43.97 ± 0.76^A^	51.85 ± 1.44^F^	54.72 ± 0.78^F^	54.46 ± 1.00^F^	57.28 ± 0.80^B^	57.81 ± 1.03^B^	58.33 ± 0.89^B^	
Bone Mineral Density (g/cm^2^)	Male	0.13 ± 0.002^A^	0.13 ± 0.002^A^	0.13 ± 0.004^A^	0.17 ± 0.003^B^	0.17 ± 0.003^B^	0.17 ± 0.003^B^	0.19 ± 0.003^C^	0.20 ± 0.004^C^	0.19 ± 0.003^C^	0.20 ± 0.001^C^	0.20 ± 0.001^C^	0.20 ± 0.002^C^	Sex: M > F Age: P50 < P80 < P140, P200 Adolescent Exposure: None
	Female	0.13 ± 0.002^A^	0.13 ± 0.003^A^	0.13 ± 0.002^A^	0.16 ± 0.003^D^	0.16 ± 0.005^D^	0.17 ± 0.003^D^	0.18 ± 0.002^E^	0.18 ± 0.001^E^	0.18 ± 0.002^E^	0.19 ± 0.003^E^	0.18 ± 0.002^E^	0.18 ± 0.002^E^	
Lean Mass (g)	Male	240.55 ± 3.51^A^	248.24 ± 5.63^A^	240.87 ± 4.56^A^	350.40 ± 6.92^B^	355.63 ± 8.65^B^	360.06 ± 7.02^B^	437.81 ± 9.00^C^	444.32 ± 12.55^C^	450.52 ± 8.58^C^	458.54 ± 10.36^D^	469.60 ± 12.91^D^	478.23 ± 9.11^D^	Sex: M > F Age: P50 < P80 < P140 < P200 Adolescent Exposure: None
	Female	166.79 ± 4.13^E^	164.32 ± 2.14^E^	165.69 ± 2.09^E^	215.79 ± 4.38^F^	215.14 ± 4.27^F^	215.08 ± 5.55^F^	262.11 ± 5.44^G^	265.74 ± 4.85^G^	258.42 ± 5.33^G^	276.61 ± 4.49^H^	267.43 ± 11.49^H^	271.55 ± 5.19^H^	
Lean Mass + Bone Mineral Content (g)	Male	246.23 ± 3.56^A^	254.15 ± 5.75^A^	246.53 ± 4.67^A^	360.23 ± 7.05^B^	365.67 ± 8.72^B^	369.84 ± 7.11^B^	451.27 ± 9.20^C^	457.94 ± 12.82^C^	464.13 ± 8.80^C^	473.19 ± 10.45^D^	484.59 ± 13.10^D^	593.37 ± 9.31^D^	Sex: M > F Age: P50 < P80 < P140 < P200 Adolescent Exposure: None
	Female	171.60 ± 4.23^E^	169.16 ± 2.16^E^	170.57 ± 2.12^E^	223.19 ± 4.43^F^	222.32 ± 4.41^F^	222.34 ± 5.55^F^	271.79 ± 5.58^G^	275.64 ± 4.92^G^	268.27 ± 5.44^G^	287.25 ± 4.54^H^	278.04 ± 11.50^H^	282.22 ± 5.24^H^	

### Experiment 1: ontogenetic changes in whole blood composition

Very few studies have investigated shifts in blood cell composition across development in the rat. Given the wide array of maturational changes that occurs during adolescence, it seemed plausible this period of change may also extend to blood leukocyte composition. Consequently, Experiment 1 assessed whole blood composition metrics using a complete blood count (CBC) in three subsets of rats in early adolescence (P28), mid adolescence (P45), and early adulthood (P90) males and females using a 2 (Sex) x 3 (Age) between-subjects factorial design (n = 8–18 per group; N = 68) (Supplementary Figure S1). Animals were euthanized with i. p. sodium pentobarbital injection, and blood samples were collected using a cardiac puncture into EDTA-containing vacutainers. These samples were immediately inverted gently to allow distribution of the EDTA into the blood sample, and then samples were placed on ice for transport to the Cornell Animal Health Diagnostic Center where they were analyzed using an ADVIA 2120 Hematology Analyzer. Animal blood composition was then analyzed for total red blood cell counts, hemoglobin, hematocrit, mean corpuscular volume (the average red blood cell size), mean corpuscular hemoglobin (the average amount of hemoglobin in each red blood cell), red blood cell distribution width, platelet counts (1,000 cells/μL blood volume), mean platelet volume, white blood cell counts (1,000 cells/μL blood volume), and absolute (1,000 cells/μL blood volume) counts and percentages of neutrophils, eosinophils, monocytes, and lymphocytes. Analysis of this data set was run using a 2 (Sex) x 3 (Age) between subjects ANOVA.

### Experiment 2: AIE effects on blood cytokine expression

Previously, we reported that adult male rats (but not females) with a history of AIE showed decreased leukocyte derived cytokine expression in response to LPS challenge (Vore et al., 2017). This finding suggests that AIE could permanently alter cytokine levels during adolescence. Consequently, this experiment initially assessed blood cytokine expression in male and female subjects before (P29), during (P44), and after (P73) adolescent exposure to either water or ethanol (AIE) as well as in non-exposed animals of the same ages using a 2 (Sex) x 3 (Adolescent Exposure) x 3 (Age) factorial design, with Age treated as a repeated measure (n = 10 per group; N = 60) (Figure 1A). Tail blood samples were collected and utilized for blood pellet PCR (Vore et al., 2017) to assess changes in blood cytokine expression across age and its interaction with AIE history. Gene expression PCR was investigated as cytokine gene expression signatures tend to be more stable than measures of cytokine proteins which have very short half-lives and are more vulnerable to degradation during freeze-thaw cycles. Separate 3 (Adolescent Exposure) x 3 (Age) mixed ANOVAs were run for each sex.

The second part of this experiment sought to assess effects of AIE on leukocyte cytokine reactivity to adult ethanol challenge. Consequently, on P74 (Ethanol Challenge) all rats received a 2.0 g/kg i. p. injection (this route of administration was chosen to produce a more profound, predictable blood ethanol concentration) of a 20% ethanol solution, and 2 hours later blood was collected and differences in cytokine expression relative to the P73 (Baseline) timepoint were evaluated. Separate 2 (Adolescent Exposure) x 2 (Ethanol Challenge) mixed ANOVAs were run for each sex.

The final portion of this experiment sought to investigate whether any AIE-associated changes in leukocyte cytokine levels could be due to altered leukocyte populations in rats with an AIE history. On P78, after a brief washout period, all rats were euthanized, and blood samples were collected using a cardiac puncture into EDTA-containing vacutainers. The samples were then transported on ice to the Cornell Animal Health Diagnostic Center and analyzed using an ADVIA 2120 Hematology Analyzer. Analysis of this data set was run using a 2 (Sex) x 3 (Age) between subjects ANOVA.

### Experiment 3: different AIE models and changes in adult cytokine reactivity to ethanol challenge

Across the field, a wide range of ethanol exposure procedures are utilized to model binge drinking during adolescence. While some aspects of these procedures may be shared (e.g., achieved BECs, intermittency, *etc.*), there are many procedural differences (e.g., schedule of exposure, total number of exposures, route of administration) that can make it difficult to replicate and interpret findings from different studies. This experiment investigated whether different models of adolescent ethanol exposure would yield similar results when evaluating the same dependent variables. To this end, collaborators at Duke University (Duke; Durham, NC) and the Medical University of South Carolina (MUSC; Charleston, SC) ran parallel studies when ethanol during adolescence was administered either by intragastrical intubation (Duke) or through inhalation of ethanol vapor (MUSC). The design of this study was a 2 (Sex) x 2 (Adolescent Exposure) x 2 (Adult Challenge) factorial. Leukocyte derived cytokine expression was then assessed on P73 (Baseline, No Challenge), after an extended period of abstinence from alcohol, and on P74 (Ethanol Challenge) 2 h following a 2.0 g kg i.p. ethanol injection.

#### Experiment 3 site protocols

Sprague-Dawley dams with pups were obtained by each institution from Envigo (Indianapolis, IN). Dams were shipped with 8 (Duke) or 10 (MUSC) male and female pups that were P15 upon arrival. After acclimation to the vivarium, pups were weaned on P21 and pair-housed with same sex littermates. Rats were maintained on a 12 h/12 h reverse light/dark cycle (lights on at 1900 h Duke and 2,100 h MUSC) with *ad libitum* access to food (MUSC: Teklab Global 18% Protein Rodent Diet 2,918, Envigo, Madison, WI; Duke: Laboratory Rodent Chow 5,001, Purina, St Hubert, Quebec) and water and remained pair-housed for the duration of the study. At the time of weaning, rats were randomly assigned to either an experimental or control group, and male/female and control/experimental groups were counterbalanced and run in tandem as separate cohorts of animals.

#### Duke and MUSC AIE exposures

Both adolescent exposure procedures involved 10 episodes of ethanol exposure with the first exposure on P28 and the last on P44, corresponding with early and late adolescence, respectively (Spear, 2015). The exposures were separated into 5 cycles, with each cycle consisting of 2 consecutive days of ethanol exposure followed by 1 non-exposure day between cycles 1 and 2 as well as between cycles 3 and 4, with 2 non-exposure days between cycles 2 and 3 as well as cycles 4 and 5 (Figure 3A). Intragastric AIE exposure (Duke) involved a well-characterized procedure in which rats were administered a dose of 5 g/kg ethanol (35% v/v in water at 18.12 mL/kg) or isovolumetric water (control) at 1,000 h on each exposure day (Risher et al., 2015). Ethanol exposure by vapor inhalation (MUSC) also involved a well-characterized procedure in which rats in standard polycarbonate housing cages (with bedding, food and water) were placed into clear acrylic chambers containing vaporized ethanol (Gass et al., 2014). Each vapor exposure period consisted of 14 h in the ethanol chambers followed by 10 h out of the chambers. Rats were placed into the chambers with same-sex littermates at 1800 h and removed at 0800 h. Intragastric AIE exposure resulted in higher BECs than exposure by vapor inhalation (200–250 mg/dL *versus* 125–190 mg/dL), with no differences evident between males and females (Healey et al., 2020). Separate 2 (Adolescent Exposure) x 2 (Ethanol Challenge) mixed ANOVAs were run for each sex.

### Experiment 4: AIE-induced changes in whole body composition

While a significant body of literature has reported the impact of alcohol on body composition in humans, far fewer studies have looked longitudinally at the impact of AIE on metrics of fat and bone mineral density in rodents. This experiment assessed whether AIE would produce changes in whole body composition that would linger past the initial alcohol exposure into later adulthood (after a long period of abstinence). Male and female rats were exposed to water, AIE, or left non-exposed. Beginning on P50, body composition was analyzed using a Hologic Discovery QDR 4500 Dual X-Ray Absorptometry Scanner. To accomplish this, rats were briefly anesthetized using dexmedetomidine (0.1 mg/kg s. c.), scanned using the DXA Scanner, and reversed using atipamezole (1.0 mg/kg i. p.). Rats under anesthesia were always kept on heating pads and with artificial tears utilized to prevent corneal damage. This procedure was then repeated at P80, P140, and P200 and changes in bone mineral content, bone mineral density, area, lean mass + bone mineral content, fat mass, total mass, and percent fat assessed (Figure 4A). Consequently, the design of this experiment was a 2 (Sex) x 3 (Adolescent Exposure) x 4 (Age) factorial, with Age treated as a repeated measure (n = 10 per group; N = 60). Analysis of this data set was run using a 2 (Sex) x 3 (Adolescent Exposure) x 4 (Age) mixed ANOVA.

## Results

### Experiment 1: ontogenetic changes in whole blood composition

#### Red blood cell metrics

A significant main effect of age on red blood cell quantity was observed (F_(2,52)_ = 128.1; *p* < 0.0001), with RBC quantities increasing across age (Table 1, Supplementary Table S2). A significant sex × age interaction (F_(2, 52)_ = 6.141; *p* = 0.004) was also noted. Male rats at P90 had significantly higher RBC quantities than their female counterparts at that age (Table 1, Supplementary Table S2). Significant main effects of sex and age were also noted on hemoglobin concentrations and hematocrit percentages. In both cases, male rats had significantly higher hemoglobin concentrations (F_(1, 52)_ = 5.425; *p* = 0.0238) and hematocrit percentages (F_(1, 52)_ = 10.90; *p* = 0.0017) than females (Table 1, Supplementary Table S2). Both hemoglobin (F_(2, 52)_ = 85.78; *p* < 0.0001) and hematocrit (F_(2, 52)_ = 27.65; *p* < 0.0001 also showed significant increases across age (Table 1, Supplementary Table S2). Finally, a significant sex × age interaction was observed on both hemoglobin levels (F_(2, 52)_ = 10.04; *p* = 0.0002) and hematocrit percentages (F_(2, 52)_ = 3.680; *p* = 0.0320), with male rats at P90 showing significantly higher levels than female counterparts at that age (Table 1, Supplementary Table S2). Significant main effects of both sex and age were also observed on both mean corpuscular volume and mean corpuscular hemoglobin. Male rats showed significantly higher MCV (F_(1, 52)_ = 14.69; *p* = 0.0003) and MCH (F_(1, 52)_ = 4.403; *p* = 0.0408) than female rats (Table 1, Supplementary Table S2). In addition, both MCV (F_(2, 52)_ = 127.4; *p* < 0.0001) and MCH (F_(2, 52)_ = 47.54; *p* < 0.0001) significantly decreased across age (Table 1, Supplementary Table S2). Significant main effects of sex and age were also noted on MCHC, with female rats showing significantly higher MCHC relative to male comparators (F_(1, 52)_ = 6.564; *p* = 0.0133) and overall levels increasing significantly with age (F_(2, 52)_ = 39.89; *p* < 0.0001) (Table 1, Supplementary Table S2). Significant main effects of sex (F_(1, 52)_ = 7.091; *p* = 0.0103) and age (F_(2, 52)_ = 201.5; *p* < 0.0001) were also observed on RBC Distribution width (Table 1, Supplementary Table S2). Male rats showed significantly higher RBC distribution width than females, and a significant decrease was observed across age.

#### Platelet metrics

A significant main effect of both sex (F_(1, 52)_ = 9.222; *p* = 0.0037) and age (F_(2, 52)_ = 12.52; *p* < 0.0001) was observed on absolute platelet quantities. Male rats showed significantly increased platelet levels relative to female rats, and platelet levels showed a significant elevation at P45 relative to both P28 and P90 (Table 1, Supplementary Table S2). A significant main effect of age (F_(2, 52)_ = 58.98; *p* < 0.0001) on mean platelet volume was also noted, platelet volume was significantly increased at P28 relative to P45 and P90 (Table 1, Supplementary Table S2).

#### White blood cell metrics

A significant main effect of age (F_(2, 52)_ = 3.348; *p* = 0.0429) was observed, with white blood cell levels being significantly decreased at P90 relative to P28 and P45 (Table 1, Supplementary Table S2). A significant main effect of sex on absolute neutrophil levels was also observed (F_(1, 52)_ = 5.802; *p* = 0.0196), with males showing significantly increased levels relative to females (Table 1, Supplementary Table S2). A significant sex × age interaction was observed on percentage of neutrophils (F_(2, 52)_ = 7.372; *p* = 0.0015), with males showing significantly higher neutrophil levels than female at P90 (Table 1, Supplementary Table S2). A significant main effect of age on percentage of eosinophils was also noted (F_(2, 53)_ = 4.787; *p* = 0.0123), with eosinophil percentages being significantly higher at P90 than at P28 or P45 (Table 1, Supplementary Table S2). Significant main effects of both sex (F_(1, 53)_ = 9.235; *p* = 0.0037) and age (F_(2, 53)_ = 16.16; *p* < 0.0001) on absolute monocyte levels were noted, with males showing significantly higher monocyte levels than females and overall monocyte levels decreasing across age (Table 1, Supplementary Table S2). Significant main effects of both sex (F_(1, 52)_ = 6.915; *p* = 0.0112) and age (F_(2, 52)_ = 18.67; *p* < 0.0001) on percentage of monocytes were also noted, with male rats showing significantly higher monocyte levels than females and overall monocyte percentages decreasing across age (Table 1, Supplementary Table S2). Absolute lymphocyte levels differed as a function of age (F_(2, 52)_ = 3.524; *p* = 0.0367), with lymphocyte levels being significantly lower at P90 than on P28 and P45 (Table 1, Supplementary Table S3). A significant main effect of sex (F_(1, 52)_ = 6.253; *p* = 0.0156) and a significant sex × age interaction (F_(2, 52)_ = 8.867; *p* = 0.0005) were observed on percentage of lymphocytes (Table 1, Supplementary Table S2). Females showed significantly higher lymphocyte percentages than males and male rats had significantly lower lymphocyte percentages than female rats at a P90 respectively (Table 1, Supplementary Table S2).

### Experiment 2: AIE effects on blood cytokine expression

#### Cytokine expression across age

Both male (F_(2,54)_ = 105.45; *p* < 0.0001) and female (F_(2,54)_ = 53.72; *p* < 0.0001) rats displayed significant main effects of age on *Il-1β* expression (Figures 1B, C; Supplementary Table S3). In both sexes, *Il-1β* levels increased with age being significantly lower at P29 than at P44 and at P44 significantly lower than at P73. Similarly, main effects of age on *Tnf-α* were observed in both male (F_(2,54)_ = 101.20; *p* < 0.0001) and female (F_(2,54)_ = 41.68; *p* < 0.0001) rats (Figure 1D, E; Supplementary Table S3). In both sexes, *Tnf-α* levels increased with age being significantly lower at P29 than at P44 and at P44 being significantly lower than at P73. Male rats showed a significant main effect of both age (F_(2,54)_ = 60.20; *p* < 0.0001) and adolescent exposure (F_(2,27)_ = 3.59; *p* = 0.04) on *Il-6* expression (Figure 1F; Supplementary Table S3). Again, *Il-6* expression levels increased across age, with levels at P29 significantly lower than at P44 which was significantly lower than expression at P73. In addition, water-exposed animals showed significantly lower *Il-6* expression than both non-exposed controls and AIE-exposed rats. A significant main effect of age (F_(2,54)_ = 35.62; *p* < 0.0001) on *Il-6* expression was also noted in female rats, with P29 *Il-6* expression being significantly lower than expression on P44 and P73 (Figure 1G; Supplementary Table S3). Similarly, main effects of age on *Iκbα* were observed in both male (F_(2,54)_ = 203.00; *p* < 0.0001) and female (F_(2,54)_ = 85.02; *p* < 0.0001) rats (Figures 1H, I; Supplementary Table S3). In both sexes, *Iκbα* levels showed an ontogenetic increase, with levels at P29 being significantly lower than at P44 and at P44 being significantly lower than at P73.

#### Cytokine reactivity to P74 ethanol challenge

A significant main effect of adolescent exposure (F_(2,27)_ = 4.59; *p* = 0.02) on *Il-1β* expression was noted in male rats (Figure 2A; Supplementary Table S4). Water-exposed controls displayed significantly lower *Il-1β* than non-exposed and AIE-exposed males. A significant main effect of adolescent exposure (F_(2,27)_ = 3.44; *p* < 0.05) on *Il-1β* expression was also observed in female rats (Figure 2B; Supplementary Table S4), with both water- and AIE-exposed showing significantly higher *Il-1β* expression than non-exposed control females. A significant main effect of ethanol challenge on *Tnf-α* was observed in both male (F_(1,27)_ = 20.04; *p* = 0.0001) and female (F_(1,27)_ = 5.07; *p* = 0.03) rats (Figures 2C, D; Supplementary Table S4). Ethanol challenge significantly decreased *Tnf-α* levels relative to baseline. A significant main effect of ethanol challenge on *Il-6* was observed in both male (F_(1,27)_ = 81.03; *p* < 0.0001) and female (F_(1,27)_ = 43.42; *p* < 0.0001) rats (Figures 2E, F; Supplementary Table S4). Ethanol challenge significantly decreased *Il-6* levels relative to baseline. Finally, a significant main effect of ethanol challenge on *Iκbα* gene expression was also noted in female rats (F_(1,27)_ = 7.15; *p* = 0.01), with ethanol challenge significantly decreasing *Iκbα* expression relative to baseline (Figure 2H; Supplementary Table S4).

#### Blood ethanol concentrations

Male rats showed no significant differences in blood ethanol concentrations after ethanol challenge in non-exposed control (144.88 ± 2.69), water-exposed control (150.09 ± 3.02), or AIE-exposed (141.41 ± 2.65) groups (Table 2, Supplementary Table S5). In female rats, no significant differences in blood ethanol concentrations after ethanol challenge were noted in non-exposed control (119.70 ± 4.22), water-exposed (115.70 ± 2.60), or AIE-exposed (113.64 ± 3.69) groups (Table 2, Supplementary Table S5).

### Whole blood composition

#### Red blood cell metrics

Significant main effects of sex were observed in RBC levels (F_(1, 46)_ = 33.51; *p* < 0.0001), hemoglobin concentrations (F_(1, 46)_ = 56.54; *p* < 0.0001), and hematocrit levels (F_(1, 46)_ = 41.25; *p* < 0.0001) (Table 3, Supplementary Table S6). Male rats showed significantly increased levels of RBC, hemoglobin, and hematocrit than females.

#### White blood cell metrics

Significant main effects of sex were observed on absolute neutrophil levels (F_(1, 46)_ = 15.26; *p* = 0.0003), and percent neutrophil (F_(1, 46)_ = 7.47; *p* = 0.009) (Table 3, Supplementary Table S6), with male rats showing significantly higher levels than females.

### Experiment 3: different AIE models and changes in adult cytokine reactivity to ethanol challenge

#### Long-term changes in cytokine reactivity to ethanol challenge after adolescent vapor exposure

A significant adolescent exposure x ethanol challenge interaction (F_(1,18)_ = 6.43; *p* = 0.02) on *Il-1β* expression was noted in male rats (Figure 3B; Supplementary Table S7). Male rats with a history of AIE displayed significantly lower *Il-1β* expression relative to air-exposed controls at baseline that was not observed following ethanol challenge. A significant main effect of ethanol challenge on *Tnf-α* was observed in males (F_(1,18)_ = 6.90; *p* = 0.02) (Figure 3D; Supplementary Table S7), with ethanol challenge significantly decreasing *Tnf-α* levels relative to baseline. A significant main effect of adolescent exposure on *Tnf-α* was observed in female rats (F_(1,18)_ = 7.91; *p* = 0.01) (Figure 3E; Supplementary Table S7). Female rats with a history of AIE displayed significantly higher *Tnf-α* levels relative to air-exposed controls. A significant adolescent exposure x ethanol challenge interaction on *Il-6* was observed in female (F_(1,18)_ = 5.31; *p* = 0.03) rats (Figure 3G; Supplementary Table S7). A significant increase in *Il-6* after ethanol challenge evident in air-exposed controls, was not observed in females with an AIE history. Finally, a significant main effect of ethanol challenge on *Iκbα* gene expression was also noted in female rats (F_(1,18)_ = 13.90; *p* = 0.002), with ethanol challenge significantly increasing *Iκbα* expression relative to baseline (Figure 3I; Supplementary Table S7).

#### Long-term changes in cytokine reactivity to ethanol challenge after intragastric AIE exposure

A significant main effect of adolescent exposure on *Tnf-α* was observed in female (F_(1,15)_ = 6.59; *p* = 0.02) rats (Figure 3M; Supplementary Table S8). Females with a history of AIE showed significantly lower *Tnf-α* levels than water-exposed control females. A significant main effect of ethanol challenge on *Il-6* was observed in both male (F_(1,15)_ = 12.63; *p* = 0.003) and female (F_(1,15)_ = 11.00; *p* = 0.005) rats (Figures 3N, O; Supplementary Table S8). Ethanol challenge significantly decreased *Il-6* levels relative to baseline.

### Experiment 4: AIE-induced changes in whole body composition

#### Body weights

While no significant effects of adolescent exposure were noted on body weights during AIE, a significant main effect of sex (F_(1,54)_ = 212.89; *p* < 0.0001), age (F_(3,162)_ = 7,335.08; *p* < 0.0001), and a significant sex × age interaction (F_(3,162)_ = 432.35; *p* < 0.0001) were observed (Figure 4B). Female rats weighed significantly less than males, with body weight increasing across age. A significant main effect of sex (F_(1,54)_ = 1808.87; *p* < 0.0001), age (F_(3,162)_ = 1334.04; *p* < 0.0001), and a sex × age interaction (F_(3,162)_ = 161.91; *p* < 0.0001) on animal weights taken across the scanning procedure was also observed (Figure 4C). Again, female rats weighed significantly less than males as weight increased with age.

#### DXA scanner metrics

Significant main effects of sex (F_(1,54)_ = 2096.20; *p* < 0.0001) and age (F_(3,162)_ = 26.61; *p* < 0.0001) as well as a significant sex × age interaction (F_(3,162)_ = 137.35; *p* < 0.0001) were observed on total mass (Figure 4D; Supplementary Table S9). Female rats weighed significantly less than males as weight increased across age. Significant main effects of sex and age as well as a significant sex × age interaction were also noted on bone mineral content [(F_(1,54)_ = 918.02; *p* < 0.0001); (F_(3,162)_ = 2775.33; *p* < 0.0001); (F_(3,162)_ = 135.96; *p* < 0.0001)], area [(F_(1,54)_ = 836.74; *p* < 0.0001); (F_(3,162)_ = 706.40; *p* < 0.0001); (F_(3,162)_ = 40.17; *p* < 0.0001)], bone mineral density [(F_(1,54)_ = 36.87; *p* < 0.0001); (F_(3,162)_ = 700.79; *p* < 0.0001); (F_(3,162)_ = 3.43; *p* = 0.02)], and lean mass + bone mineral content [(F_(1,54)_ = 1654.77; *p* < 0.0001); (F_(3,162)_ = 865.49; *p* < 0.0001); (F_(3,162)_ = 105.14; *p* < 0.0001)] (Table 4, Supplementary Table S9). A significant main effect of sex (F_(1,54)_ = 10.96; *p* = 0.002) and age (F_(3,162)_ = 26.61; *p* < 0.0001) as well as a sex x adolescent exposure interaction (F_(2,54)_ = 3.19; *p* = 0.049) on fat mass were observed (Figure 4G; Supplementary Table S9). Again, female rats had significantly lower fat mass levels than male comparators and fat mass increased with age. However, only AIE-exposed male rats had significantly lower fat mass than water-exposed controls. Finally, a significant sex x adolescent exposure interaction (F_(2,54)_ = 3.31; *p* = 0.044) on percent fat was also observed (Figure 4F; Supplementary Table S9). Male rats with a history of AIE showed significantly reduced body fat percentages relative to AIE-exposed females.

## Discussion

The present studies tested the hypothesis that AIE would produce long-lasting changes in body and blood composition that might contribute to durable alterations in circulating cytokine expression. Experiment 1 revealed age-dependent shifts in WBC quantities and leukocyte levels that tend to replicate human data (Li et al., 2020), emphasizing the importance of considering natural age differences in growth and physiology before evaluation of how experiential factors such as ethanol exposure influence the trajectory of natural aging. While total WBC levels, monocyte, and lymphocyte quantities decreased with age, neutrophils and eosinophils increased as animals reached P90. These alterations in total leukocyte levels likely contribute to natural shifts in immunity observed with age and suggest that throughout adolescence, alterations in leukocyte differentiation could contribute to enhanced adolescent vulnerability to alcohol-induced alterations. Subsequently, Experiment 2 revealed age-related changes in leukocyte derived cytokine expression. Expression levels of almost all cytokine targets were very low at P29 and increased steadily into adulthood. However, AIE had no effects on the majority of these targets under basal or ethanol challenge conditions. No AIE-induced changes in white blood cell counts were evident as well, suggesting that despite the high levels of BECs achieved and exposure frequency, rat blood composition remained normal.

Experiment 3 revealed that while ethanol challenge in adulthood reduced *Il-6* expression, there was no effect of intragastric AIE on *Il-6*. However, intragastric AIE significantly reduced *Tnf-α* levels in female rats. In contrast, AIE exposure through vapor inhalation produced markedly different patterns of cytokine expression. A significant reduction in *Il-1β* expression in male rats with an AIE history relative to air-exposed controls at baseline was observed. Female rats with a history of AIE also showed significantly elevated *Tnf-α* levels relative to air-exposed control females regardless of challenge. Control female rats exposed to air during adolescence showed a significant increase in *Il-6* expression in response to ethanol challenge, with this ethanol effect not observed in females with a history of AIE exposure through vapor inhalation. Finally, female rats showed a significant increase in *Iκbα* expression in response to ethanol challenge. These differences between intragastric and inhalation routes of AIE exposure highlight the increasing need to cross validate different models of AIE and suggest that route of administration rather than AIE in some cases may drive reported changes.

Finally, Experiment 4 revealed that male and female rats with a history of AIE show divergent patterns of fat accumulation across the life span. Male rats with a history of AIE show significantly reduced fat mass levels relative to water-exposed controls and male rats with a history of AIE show significantly lower % fat levels than female rats with a history of AIE. These alterations appear to begin shortly after AIE (between P50 and P80) and persist well into adulthood (P200). Despite this, no long-term changes in bone integrity or lean mass were noted, suggesting a highly specific effect of AIE on adiposity that may continue even further across the lifespan.

Very few studies have characterized the developmental trajectory of differential white blood cell counts, particularly in rats. Our study revealed multiple age-related differences in total white blood cell composition. These findings suggest that multiple physiological maturational changes during adolescence also include maturation and shift for leukocytes. As a significant player in orchestration and propagation of the peripheral immune response, these findings contribute to a growing body of work focused on immune development and the concept of a functionally immature immune response earlier in life (Simon, Hollander and McMichael, 2015; Georgountzou and Papadopoulos, 2017). This notion is further supported by the leukocyte cytokine patterns observed in Experiment 2, with all cytokine targets displaying a large reduction in early adolescence and followed by an increase in adulthood. Given that no significant changes resulting from AIE were seen in either cytokine expression or leukocyte cell differentials, the observed age differences likely reflect functional alterations within the leukocytes or other immune signaling molecules. The low levels of IκBα could reflect altered NFκB signaling in circulating monocyte and lymphocyte populations. Individuals with early-onset bipolar disorder and Major Depressive Disorder (MDD) both show high basal levels of NFκB in adolescence that correlate with higher levels of circulating cytokines and individuals with early-onset bipolar disorder and MDD (Miklowitz et al., 2016). NFκB is known to play a role in complex behavioral responses outside of traditional immunity (Nennig and Schank, 2017), and it is possible that a developmental shift in NFκB-responsive elements could play a functional role in the synaptic pruning associated with normal adolescent brain maturation (Socodato et al., 2020).

While no long-term AIE-associated changes in basal or ethanol evoked cytokine gene expression were observed, this finding replicates our previously reported results with ethanol used as a challenge stimulus (Vore et al., 2017). Previously, we have shown a long-lasting attenuation of peripheral cytokine gene expression in male rats with a history of AIE but only in response to adult challenge by either restraint or LPS. Particularly regarding LPS challenge, this finding may suggest that AIE can produce long-lasting alterations of peripheral immune function evident primarily after canonical immune challenge, such as bacterial mimetics, as well as centrally mediated host defense processes such as fever regulation (Telles et al., 2017; Cruz et al., 2020; see Deak et al., 2022 for review). As CBC lacks the capacity to detect functional shifts in lymphocyte or monocyte subpopulations, future studies utilizing flow cytometry would allow for more extensive characterization of AIE-induced alterations of leukocyte immunoreactivity independent of total number changes. Indeed, much of the existing work examining alcohol effects on blood cells has shown effects on monocyte phenotype (Janicova et al., 2021) or has investigated changes in human subjects with a history of AUD. These studies, while very informative, are often complicated by the poor diet (Cummings et al., 2020), sedentary behavior, and other high-risk behaviors engaged by individuals with AUD, suggesting that adolescent alcohol exposure alone may not be the driving cause for many of the hematological complications associated with heavy alcohol use.

Given the diverse array of AIE models, there is a growing concern in the field regarding generalizability of main findings among these models. The lack of AIE effects on ethanol evoked cytokine expression in adulthood largely replicates our previous findings (Vore et al., 2017). In addition, Experiment 3 together with Experiment 2 provided a unique opportunity to directly compare three models of AIE exposure using the same dependent variables. Although our intragastric AIE model (see Experiment 2) and the intragastric model of AIE in Experiment 3 utilized different ethanol doses, number of exposures, and schedules of ethanol exposure, both intragastric AIE models produced very few changes in basal blood cytokine expression; 5 g/kg i. g exposure producing a modest decrease in *Tnf-α* under basal and ethanol challenge conditions in females. In contrast, vapor exposure to alcohol significantly reduced baseline *Il-1β* in males and increased *Tnf-α* under basal and ethanol challenge conditions in females. Vapor exposure models are frequently used to model alcohol dependence in laboratory rodents (Becker and Lopez, 2004; Gilpin et al., 2008). The longer and more consistent periods of intoxication associated with these models may more readily produce durable changes in cytokines but may not reflect less intense binge consumption. It is also possible that the differences observed between route of administration are the result of effects of gavage on cytokine expression. Experiment 2 revealed significant differences between gavage and unmanipulated controls in *Il-1β* expression in both sexes. These differences could interact with AIE and alter cytokine expression in a disparate manner from vapor exposure. In addition, alcohol delivered directly into the stomach via gavage could directly alter gut microflora in a manner distinct from vapor exposure models. Alcohol has been shown to contribute to dysbiosis of the gut microbiome (Jew and Hsu, 2023) which can increase endotoxin release and heighten peripheral inflammation. This difference between models could readily change cytokine levels both during developmental exposure as well as in response to adult challenge. Contributing to the scientific rigor of these experiments is the inclusion of both sexes as well as the incorporation of non-exposed controls into Experiments 2 and 4.

Beyond sex differences in the prevalence of AUD and treatment outcomes (Agabio et al., 2017), sex differences exist in ethanol metabolism (Thomasson et al., 1995), behavioral consequences (Jury et al., 2017), and many other aspects of alcohol use (see Flores-Bonilla and Richardson, 2020 for a recent review). This is often particularly true when investigating AIE as it overlaps with pubertal maturation, the time frame and hormonal changes associated with which differ greatly by sex (Kasten et al., 2020; Vore et al., 2021; Healey et al., 2022). Established sex differences in both adiposity (Karastergiou et al., 2012) and bone mineral density and size (Hasselstrøm et al., 2006) begin to emerge during puberty (Loomba-Abrechtt and Styne, 2009; Elhakeem et al., 2019). Therefore, we hypothesized that AIE would produce long-lasting changes in both bone density and fat. Surprisingly, despite established dose-dependent alcohol effects on bone mineral density, with lower alcohol exposure levels increasing BMD (Holbrook and Barrett-Connor, 1993; Jugdaohsingh et al., 2006) and higher ethanol exposure levels decreasing BMD (Jang et al., 2017), no significant effects of AIE on bone composition were found. Much of this work involved human subjects with AUD diagnosis, which could be confounded by factors such as diet and sedentary lifestyle alterations associated with heavy alcohol use. Our data suggest that in the absence of such confounds, AIE alone does not seem to impact bone integrity across development.

One of the more interesting results of these experiments is that AIE produced long-lasting sex divergent effects on body fat levels evident in adulthood. Sex differences in adipose tissue content, particularly in regard to obesity, are well established. In both humans and rats, females tend to have higher levels of subcutaneous adipose tissue (SAT) and males tend to have more visceral adipose tissue (VAT) which surrounds the abdominal organs (Chang, Varghese and Singer, 2018). Alcohol specifically is known to reduce adipose tissue mass as well as alter inflammation in adipose compartments, contributing greatly to the pathology of alcoholic liver disease (Kema et al., 2015). One of the most thoroughly characterized adipokines, leptin, has been identified as a key target in immune-metabolism interaction. In circumstances of both obesity (Matarese et al., 2010) and malnutrition (Cohen et al., 2017), alterations in leptin have correlated with shifts in immune function. In addition, leptin is known to have an important role in the CNS and specifically in modulating the HPA axis response at the level of the hypothalamus (Pérez-Pérez et al., 2017). Alcohol consumption in males has previously been shown to decrease leptin levels (Röjdmark et al., 2001; Otaka et al., 2007), and leptin has been suggested to be involved in both the formation of alcoholic fatty liver disease (Tan et al., 2012) as well as craving/relapse (Aguiar-Nemer et al., 2013; Haass-Koffler et al., 2015). Lower fat levels and lowered leptin levels seen in male rats after alcohol exposure (Otaka et al., 2007), could significantly blunt the immune response to future challenge (Francisco et al., 2018).

Though the present series of experiments reflect an extremely thorough, well controlled investigation of maturational changes across adolescent development and the long-term consequences of AIE on whole body physiology, this was not intended to be a comprehensive assessment of immune function. While CBC is an effective method for analyzing gross changes in whole blood composition, functional profiling of immune cell subsets using a technique such as flow cytometry could reveal more complex shifts in immune reactivity as a result of AIE that were missed with CBC. Likewise, all of the cytokine data presented reflects alterations in gene expression and could have missed alterations in functional cytokine protein release. Cytokines are typically released locally and highly transiently and as such, it is common to use cytokine mRNA as a proxy for shifts in cytokine protein (Vore et al., 2017; Israelsson et al., 2020). It is also worth highlighting that significant differences existed between the three models of AIE used in Experiment 2 and Experiment 3. Differences in animals housing conditions and early life prior to exposure (for example, the animals in Experiment 2 were bred in-house whereas the animals in Experiment 3 were shipped in) could influence interpretation of the differences noted in these experiments. There are also significant differences in the kinetics of ethanol administered via intragastric gavage or through vapor exposure. Namely, vapor exposure of alcohol bypasses first pass metabolism by the stomach and liver. Since the liver fulfills an important immune role (Kubes and Jenne, 2018) it is possible that this difference could significantly affect the cytokine changes during AIE. Finally, blood ethanol concentrations were not confirmed following adult ethanol challenge in experiment 3. It is possible that the observed differences in cytokine gene expression between the two exposures could reflect changes in ethanol metabolism stemming from the different routes of administration. Despite these minor limitations, the present studies add critical new information on long-lasting physiological effects produced by AIE, with important implications for subsequent immune function and body composition.

While in these studies AIE did not alter leukocyte levels or evoked cytokine expression, it is possible that more complex phenotypic shifts would still reveal immune dysfunction in the face of bacterial or viral challenge (Vore et al., 2017). This could be further exaggerated by the durable, sex-specific alterations in fat levels observed in adult rats with a history of AIE. Fat plays an important role in modulating the fever response to viral or bacterial challenge (Pohl et al., 2014; Rummel et al., 2016). Brown adipose tissue (BAT) has specifically been highlighted as a key source of thermogenic activity that cytokines both target and are released from under conditions of bacterial infection (Liu et al., 2022). Ethanol is capable of permeating into and being detected at significant levels in BAT (Shih and Taberner, 2000) and BAT is known to express ADH though at much lower levels than what is observed in the liver (Kortelainen et al., 1991), suggesting a possible role for ethanol in BAT function. While reports of the effects of chronic ethanol on BAT have been mixed, several studies have reported that chronic ethanol consumption is capable of decreasing total BAT mass (Muraldihara and Desautels, 1996; Blaner et al., 2017). Few studies have directly measured the consequences of AIE on BAT levels, however the significantly lower levels of fat observed here in AIE-exposed male rats could reflect long-term, changes in BAT mass that would significantly hinder appropriate inflammation responses to immune challenges and may contribute to the reported heightened vulnerability observed in individuals with a history of AUD (Szabo and Saha, 2015).

## Data Availability

The raw data supporting the conclusion of this article will be made available by the authors, without undue reservation.
