# Supplementary material for: Adolescent intermittent ethanol (AIE) produces lasting, sex-specific changes in rat body fat independent of changes in white blood cell composition
Source: Front Physiol. 2024 Jan 24;15:1285376. doi: 10.3389/fphys.2024.1285376 (PMC10851431; doi:10.3389/fphys.2024.1285376)
Supplement: Supplementary file 1 [file Table1.DOCX]

**Supplemental Table 1. Sequences of Primers Used for RT-PCR Studies**

| **Gene** | **Accession Number** | **Forward Primer (5’-3’)** | **Reverse Primer (5’-3’)** |
| --- | --- | --- | --- |
| *Il-1β* | NM_031512.2 | TCCTCTGTGACTCGTGGGAT | TGGAGAATACCACTTGTTGGCT |
| *Tnf-α* | NM_012675.3 | GTCCCAACAAGGAGGAGAAGTT | CTCCGCTTGGTGGTTTGCTA |
| *Il-6* | NM_012589.2 | TAGTCCTTCCTACCCCAACTTCC | TTGGTCCTTAGCCACTCCTTC |
| *Iκbα* | NM_001105720.2 | CTGTTGAAGTGTGGGGCTGA | AGGGCAACTCATCTTCCGTG |

**Supplemental Table 2. Experiment 1: Blood Composition Analysis Across Development**

ANOVA results for all measured blood composition dependent variables in Experiment 1. Significant effects are bolded.

|  | *Sex* | *Age (P28 v P45 v P90)* | *Sex x Age* |
| --- | --- | --- | --- |
| *Red Blood Cell* | F (1, 52) = 0.7153 P=0.40 | **F (2, 52) = 128.1 P<0.0001** | **F (2, 52) = 6.141 P=0.004** |
| *Hemoglobin* | **F (1, 52) = 5.425**  **P=0.0238** | **F (2, 52) = 85.78**  **P<0.0001** | **F (2, 52) = 10.04**  **P=0.0002** |
| *Hematocrit* | **F (1, 52) = 10.90**  **P=0.0017** | **F (2, 52) = 27.65**  **P<0.0001** | **F (2, 52) = 3.680**  **P=0.0320** |
| *Mean Corpuscular Volume* | **F (1, 52) = 14.69**  **P=0.0003** | **F (2, 52) = 127.4**  **P<0.0001** | F (2, 52) = 1.194  P=0.3111 |
| *Mean Corpuscular Hemoglobin* | **F (1, 52) = 4.403**  **P=0.0408** | **F (2, 52) = 47.54**  **P<0.0001** | F (2, 52) = 0.9567  P=0.3908 |
| *MCHC* | **F (1, 52) = 6.564**  **P=0.0133** | **F (2, 52) = 39.89**  **P<0.0001** | F (2, 52) = 2.202  P=0.1208 |
| *Red Blood Cell Distribution Width* | **F (1, 52) = 7.091**  **P=0.0103** | **F (2, 52) = 201.5**  **P<0.0001** | F (2, 52) = 0.7966  P=0.4563 |
| *Absolute Platelets* | **F (1, 52) = 9.222**  **P=0.0037** | **F (2, 52) = 12.52**  **P<0.0001** | F (2, 52) = 1.360  P=0.2655 |
| *Mean Platelet Volume* | F (1, 52) = 0.001910  P=0.9653 | **F (2, 52) = 58.98**  **P<0.0001** | F (2, 52) = 0.5609  P=0.5741 |
| *WBC* | F (1, 52) = 3.264  P=0.0766 | **F (2, 52) = 3.348**  **P=0.0429** | F (2, 52) = 0.1382  P=0.8712 |
| *Absolute Neutrophil Count* | **F (1, 52) = 5.802**  **P=0.0196** | F (2, 52) = 0.3213  P=0.7266 | F (2, 52) = 2.894  P=0.0643 |
| *Percent Neutrophil* | F (1, 52) = 3.961  P=0.0518 | F (2, 52) = 1.512  P=0.2301 | **F (2, 52) = 7.372**  **P=0.0015** |
| *Absolute Eosinophil Count* | F (1, 52) = 0.06293  P=0.8029 | F (2, 52) = 2.752  P=0.0731 | F (2, 52) = 1.446  P=0.2449 |
| *Percent Eosinophil* | F (1, 53) = 0.2074  P=0.6507 | **F (2, 53) = 4.787**  **P=0.0123** | F (2, 53) = 0.9927  P=0.3774 |
| *Absolute Monocyte Count* | **F (1, 53) = 9.235**  **P=0.0037** | **F (2, 53) = 16.16**  **P<0.0001** | F (2, 53) = 0.5834  P=0.5616 |
| *Percent Monocyte* | **F (1, 52) = 6.915**  **P=0.0112** | **F (2, 52) = 18.67**  **P<0.0001** | F (2, 52) = 1.236  P=0.2989 |
| *Absolute Lymphocyte Count* | F (1, 52) = 2.497  P=0.1201 | **F (2, 52) = 3.524**  **P=0.0367** | F (2, 52) = 0.3840  P=0.6831 |
| *Percent Lymphocyte* | **F (1, 52) = 6.253**  **P=0.0156** | F (2, 52) = 0.3557  P=0.7024 | **F (2, 52) = 8.867**  **P=0.0005** |
| *Absolute Basophil Count* | N/A | N/A | N/A |
| *Percent Basophil* | N/A | N/A | N/A |

**Supplemental Table 3. Experiment 2: Cytokine Expression Across Age**

ANOVA results for cytokine gene expression measured across P29-P73 in Experiment 2. Significant effects are bolded.

|  | *Male* | | | *Female* | | |
| --- | --- | --- | --- | --- | --- | --- |
|  | *Adolescent Exposure* | *Age* | *Adolescent Exposure x Age* | *Adolescent Exposure* | *Age* | *Adolescent Exposure x Age* |
| *Il-1β* | F (2, 27) =1.30  P=0.29 | **F (2, 54) = 105.45**  **P<0.0001** | F (4, 54) = 0.73  P=0.57 | F (2, 27) =0.47  P=0.63 | **F (2, 54) = 53.72**  **P<0.0001** | F (4, 54) = 1.59  P=0.19 |
| *Tnf-α* | F (2, 27) =0.26  P=0.77 | **F (2, 54) = 101.20**  **P<0.0001** | F (4, 54) = 0.84  P=0.51 | F (2, 27) =1.42  P=0.26 | **F (2, 54) = 41.68**  **P<0.0001** | F (4, 54) = 0.79  P=0.54 |
| *Il-6* | **F (2, 27) =3.59**  **P=0.04** | **F (2, 54) = 60.20**  **P<0.0001** | F (4, 54) = 0.53  P=0.72 | F (2, 27) =2.05  P=0.15 | **F (2, 54) = 35.62**  **P<0.0001** | F (4, 54) = 0.70  P=0.60 |
| *Iκbα* | F (2, 27) =2.73  P=0.08 | **F (2, 54) = 203.00**  **P<0.0001** | F (4, 54) = 0.48  P=0.75 | F (2, 27) =2.75  P=0.08 | **F (2, 54) = 85.02**  **P<0.0001** | F (4, 54) = 1.22  P=0.31 |

**Supplemental Table 4. Experiment 2: Cytokine Reactivity to P74 Ethanol Challenge**

ANOVA results for cytokine gene expression after adult ethanol challenge (relative to P73 baseline) in Experiment 2. Significant effects are bolded.

|  | *Male* | | | *Female* | | |
| --- | --- | --- | --- | --- | --- | --- |
|  | *Adolescent Exposure* | *Ethanol Challenge* | *Adolescent Exposure x Ethanol Challenge* | *Adolescent Exposure* | *Ethanol Challenge* | *Adolescent Exposure x Ethanol Challenge* |
| *Il-1β* | **F (2, 27) =4.59**  **P=0.02** | F (1, 27) = 0.10  P=0.76 | F (2, 27) = 0.94  P=0.40 | **F (2, 27) =3.44**  **P=0.047** | F (1, 27) = 1.57  P=0.22 | F (2, 27) = 0.22  P=0.80 |
| *Tnf-α* | F (2, 27) =2.14  P=0.13 | **F (1, 27) = 20.04**  **P=0.0001** | F (2, 27) = 0.12  P=0.89 | F (2, 27) =0.77  P=0.47 | **F (1, 27) = 5.07**  **P=0.03** | F (2, 27) = 0.64  P=0.53 |
| *Il-6* | F (2, 27) =2.00  P=0.15 | **F (1, 27) = 81.03**  **P<0.0001** | F (2, 27) = 0.36  P=0.70 | F (2, 27) =0.46  P=0.63 | **F (1, 27) = 43.42**  **P<0.0001** | F (2, 27) = 0.63  P=0.54 |
| *Iκbα* | F (2, 27) =1.29  P=0.29 | F (1, 27) = 0.23  P=0.63 | F (2, 27) = 0.13  P=0.88 | F (2, 27) =1.45  P=0.25 | **F (1, 27) = 7.15**  **P=0.01** | F (2, 27) = 0.07  P=0.93 |

**Supplemental Table 5. Experiment 2: Blood Ethanol Concentrations after P74 Ethanol Challenge**

ANOVA results for blood ethanol concentrations after adult ethanol challenge in Experiment 2. Significant effects are bolded.

|  |  | *Adolescent Exposure* |
| --- | --- | --- |
| *BECs* | *Male* | F (2, 27) =2.44  P=0.11 |
|  | *Female* | F (2, 27) =0.74  P=0.48 |

**Supplemental Table 6. Experiment 2: Blood Composition Analysis**

ANOVA results for all measured blood composition dependent variables in Experiment 2. Significant effects are bolded.

|  | *Sex* | *Adolescent Exposure (Control v Water v AIE)* | *Sex x Adolescent Exposure* |
| --- | --- | --- | --- |
| *Red Blood Cell* | **F (1, 46) = 33.51**  **P<0.0001** | F (2, 46) = 0.7573  P=0.4747 | F (2, 46) = 1.733  P=0.1881 |
| *Hemoglobin* | **F (1, 46) = 56.54**  **P<0.0001** | F (2, 46) = 0.4581  P=0.6353 | F (2, 46) = 1.473  P=0.2399 |
| *Hematocrit* | **F (1, 46) = 41.25**  **P<0.0001** | F (2, 46) = 0.1316  P=0.8771 | F (2, 46) = 1.642  P=0.2048 |
| *Mean Corpuscular Volume* | F (1, 46) = 1.895  P=0.1753 | F (2, 46) = 1.595  P=0.2139 | F (2, 46) = 0.3486  P=0.7075 |
| *Mean Corpuscular Hemoglobin* | F (1, 46) = 0.3051  P=0.5834 | F (2, 46) = 2.025  P=0.1436 | F (2, 46) = 0.1080  P=0.8978 |
| *MCHC* | F (1, 46) = 1.378  P=0.2465 | F (2, 46) = 0.04413  P=0.9569 | F (2, 46) = 0.3882  P=0.6805 |
| *Red Blood Cell Distribution Width* | F (1, 46) = 2.394  P=0.1287 | F (2, 46) = 2.486  P=0.0944 | F (2, 46) = 2.471  P=0.0957 |
| *Absolute Platelets* | F (1, 46) = 0.7899  P=0.3788 | F (2, 46) = 0.6336  P=0.5353 | F (2, 46) = 1.487  P=0.2368 |
| *Mean Platelet Volume* | F (1, 46) = 1.675  P=0.2020 | F (2, 46) = 0.3188  P=0.7286 | F (2, 46) = 1.476  P=0.2392 |
| *WBC* | F (1, 46) = 4.023  P=0.0508 | F (2, 46) = 0.3114  P=0.7339 | F (2, 46) = 0.07210  P=0.9305 |
| *Absolute Neutrophil Count* | **F (1, 46) = 15.26**  **P=0.0003** | F (2, 46) = 2.098  P=0.1343 | F (2, 46) = 0.1201  P=0.8871 |
| *Percent Neutrophil* | **F (1, 46) = 7.466**  **P=0.0089** | F (2, 46) = 1.889  P=0.1628 | F (2, 46) = 1.164  P=0.3212 |
| *Absolute Eosinophil Count* | F (1, 46) = 0.4967  P=0.4845 | F (2, 46) = 0.4144  P=0.6632 | F (2, 46) = 1.951  P=0.1538 |
| *Percent Eosinophil* | F (1, 46) = 1.221  P=0.2748 | F (2, 46) = 0.2412  P=0.7867 | F (2, 46) = 0.06188  P=0.9401 |
| *Absolute Monocyte Count* | F (1, 46) = 0.6784  P=0.4144 | F (2, 46) = 0.4978  P=0.6111 | F (2, 46) = 0.3648  P=0.6963 |
| *Percent Monocyte* | F (1, 46) = 0.3403  P=0.5625 | F (2, 46) = 1.044  P=0.3604 | F (2, 46) = 1.096  P=0.3428 |
| *Absolute Lymphocyte Count* | F (1, 46) = 2.538  P=0.1180 | F (2, 46) = 0.3782  P=0.6872 | F (2, 46) = 0.06457  P=0.9376 |
| *Percent Lymphocyte* | F (1, 46) = 3.601  P=0.0640 | F (2, 46) = 1.861  P=0.1671 | F (2, 46) = 0.6579  P=0.5227 |
| *Absolute Basophil Count* | N/A | N/A | N/A |
| *Percent Basophil* | N/A | N/A | N/A |

**Supplemental Table 7. Experiment 3: Long-Term Changes in Cytokine Reactivity to Ethanol Challenge after Adolescent Vapor Exposure**

ANOVA results for cytokine gene expression in rats with an adolescent vapor exposure history after adult ethanol challenge (relative to P73 baseline) in Experiment 3. Significant effects are bolded.

|  | *Male* | | | *Female* | | |
| --- | --- | --- | --- | --- | --- | --- |
|  | *Adolescent Exposure* | *Age* | *Adolescent Exposure x Age* | *Adolescent Exposure* | *Age* | *Adolescent Exposure x Age* |
| *Il-1β* | F (1, 18) =0.31  P=0.58 | F (1, 18) = 1.43  P=0.25 | **F (1, 18) = 6.43**  **P=0.02** | F (1, 18) =3.84  P=0.07 | F (1, 18) = 3.40  P=0.08 | F (1, 18) = 0.89  P=0.36 |
| *Tnf-α* | F (1, 18) =0.11  P=0.74 | **F (1, 18) = 6.90**  **P=0.02** | F (1, 18) = 1.53  P=0.23 | **F (1, 18) =7.91**  **P=0.01** | F (1, 18) = 1.04  P=0.32 | F (1, 18) = 0.38  P=0.54 |
| *Il-6* | F (1, 18) =0.28  P=0.60 | F (1, 18) = 1.61  P=0.22 | F (1, 18) = 3.10  P=0.10 | F (1, 18) =0.03  P=0.86 | F (1, 18) = 0.82  P=0.38 | **F (1, 18) = 5.31**  **P=0.03** |
| *Iκbα* | F (1, 18) =0.66  P=0.43 | F (1, 18) = 1.00  P=0.33 | F (1, 18) = 0.70  P=0.41 | F (1, 18) =2.21  P=0.15 | **F (1, 18) =13.90**  **P=0.002** | F (1, 18) = 0.15  P=0.70 |

**Supplemental Table 8. Experiment 3: Long-Term Changes in Cytokine Reactivity to Ethanol Challenge after Intragastric AIE Exposure**

ANOVA results for cytokine gene expression in rats with an intragastric AIE exposure history after adult ethanol challenge (relative to P73 baseline) in Experiment 3. Significant effects are bolded.

|  | *Male* | | | *Female* | | |
| --- | --- | --- | --- | --- | --- | --- |
|  | *Adolescent Exposure* | *Ethanol Challenge* | *Adolescent Exposure x Ethanol Challenge* | *Adolescent Exposure* | *Ethanol Challenge* | *Adolescent Exposure x Ethanol Challenge* |
| *Il-1β* | F (1, 15) =0.25  P=0.63 | F (1, 15) = 0.003  P=0.96 | F (1, 15) = 0.007  P=0.93 | F (1, 15) =0.67  P=0.42 | F (1, 15) = 0.11  P=0.74 | F (1, 15) = 0.23  P=0.64 |
| *Tnf-α* | F (1, 15) =1.15  P=0.30 | F (1, 15) = 0.19  P=0.67 | F (1, 15) = 2.68  P=0.12 | **F (1, 15) =6.59**  **P=0.02** | F (1, 15) = 0.13  P=0.73 | F (1, 15) = 0.10  P=0.76 |
| *Il-6* | F (1, 15) =1.01  P=0.33 | **F (1, 15) = 12.63**  **P=0.003** | F (1, 15) = 0.10  P=0.75 | F (1, 15) =0.03  P=0.86 | **F (1, 15) = 11.00**  **P=0.005** | F (1, 15) = 0.001  P=0.97 |
| *Iκbα* | F (1, 15) =2.23  P=0.16 | F (1, 15) = 1.09  P=0.31 | F (1, 15) = 1.99  P=0.18 | **F (1, 15) =5.72**  **P=0.03** | F (1, 15) = 0.66  P=0.43 | F (1, 15) = 0.49  P=0.49 |

**Supplemental Table 9. Experiment 4: DXA Scanner Analyses**

ANOVA results for all measured DXA Scanner dependent variables in Experiment 4. Significant effects are bolded.

|  | *Sex* | *Adolescent Exposure* | *Age* | *Sex x Adolescent Exposure* | *Sex x Age* | *Adolescent Exposure x Age* | *Sex x Adolescent Exposure x Age* |
| --- | --- | --- | --- | --- | --- | --- | --- |
| *Bone Mineral Content* | **F (1, 54) = 918.02**  **P<0.0001** | F (2, 54) =0.56  P=0.58 | **F (3, 162) = 2775.33**  **P<0.0001** | F (2, 54) = 0.51  P=0.60 | **F (3, 162) = 135.96**  **P<0.0001** | F (6, 162) = 0.56  P=0.76 | F (6, 162) = 0.48  P=0.82 |
| *Area* | **F (1, 54) = 836.74**  **P<0.0001** | F (2, 54) =2.59  P=0.08 | **F (3, 162) = 706.40**  **P<0.0001** | F (2, 54) = 0.44  P=0.65 | **F (3, 162) = 40.17**  **P<0.0001** | F (6, 162) = 0.85  P=0.53 | F (6, 162) = 0.84  P=0.54 |
| *Bone Mineral Density* | **F (1, 54) = 36.87**  **P<0.0001** | F (2, 54) =1.30  P=0.28 | **F (3, 162) = 700.79**  **P<0.0001** | F (2, 54) = 0.44  P=0.64 | **F (3, 162) = 3.43**  **P=0.02** | F (6, 162) = 0.54  P=0.77 | F (6, 162) = 0.71  P=0.64 |
| *Lean Mass* | **F (1, 54) = 1632.65**  **P<0.0001** | F (2, 54) =0.67  P=0.52 | **F (3, 162) = 806.45**  **P<0.0001** | F (2, 54) = 1.58  P=0.22 | **F (3, 162) = 100.46**  **P<0.0001** | F (6, 162) = 0.30  P=0.94 | F (6, 162) = 0.44  P=0.85 |
| *Lean Mass +Bone Mineral Content* | **F (1, 54) = 1654.77**  **P<0.0001** | F (2, 54) =0.42  P=0.66 | **F (3, 162) = 865.49**  **P<0.0001** | F (2, 54) = 1.15  P=0.32 | **F (3, 162) = 105.14**  **P<0.0001** | F (6, 162) = 0.24  P=0.96 | F (6, 162) = 0.48  P=0.82 |
| *Fat Mass* | **F (1, 54) =10.96**  **P=0.0017** | F (2, 54) =0.20  P=0.82 | **F (3, 162) = 26.61**  **P<0.0001** | **F (2, 54) = 3.19**  **P=0.049** | F (3, 162) = 2.33  P=0.07 | F (6, 162) = 0.32  P=0.93 | F (6, 162) = 0.95  P=0.46 |
| *Total Mass* | **F (1, 54) =2096.20**  **P<0.0001** | F (2, 54) =0.61  P=0.55 | **F (3, 162) = 1184.92**  **P<0.0001** | F (2, 54) = 0.94  P=0.40 | **F (3, 162) = 137.35**  **P<0.0001** | F (6, 162) = 0.22  P=0.97 | F (6, 162) = 0.38  P=0.89 |
| *Percent Fat* | F (1, 54) =3.47  P=0.068 | F (2, 54) =0.02  P=0.98 | F (3, 162) = 1.98  P=0.12 | **F (2, 54) = 3.31**  **P=0.044** | F (3, 162) = 0.58  P=0.63 | F (6, 162) = 0.44  P=0.85 | F (6, 162) = 1.07  P=0.38 |
