# Supplementary figures and images for: Adolescent intermittent ethanol (AIE) produces lasting, sex-specific changes in rat body fat independent of changes in white blood cell composition
Source: Front Physiol. 2024 Jan 24;15:1285376. doi: 10.3389/fphys.2024.1285376 (PMC10851431; doi:10.3389/fphys.2024.1285376)

## Slide 1
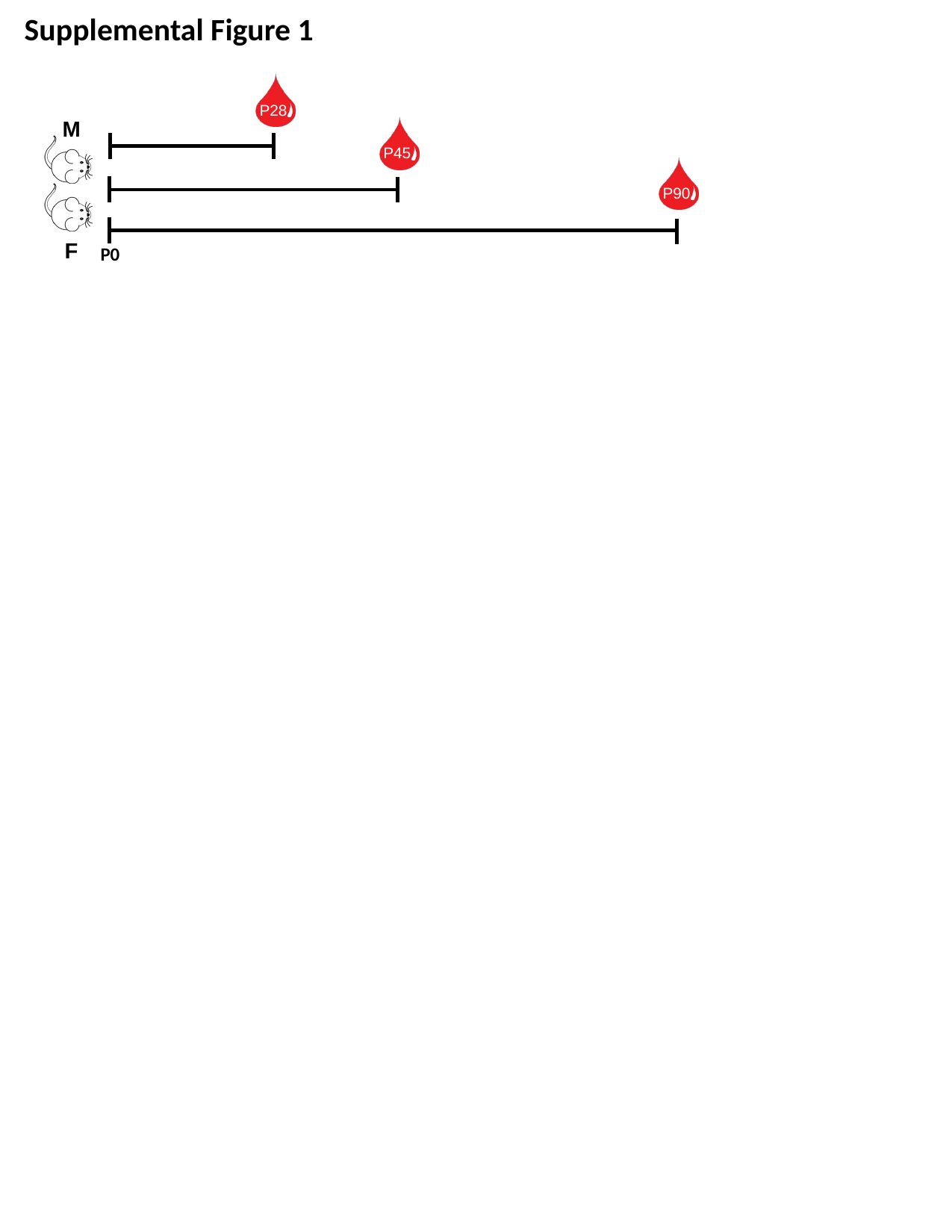

Supplemental Figure 1
P28
M
P45
P90
F
P0

Supplement: Supplementary file 2 [file Presentation1.PPTX]
